# Metabolomics Suggests That Soil Inoculation with Arbuscular Mycorrhizal Fungi Decreased Free Amino Acid Content in Roots of Durum Wheat Grown under N-Limited, P-Rich Field Conditions

**DOI:** 10.1371/journal.pone.0129591

**Published:** 2015-06-11

**Authors:** Sergio Saia, Paolo Ruisi, Veronica Fileccia, Giuseppe Di Miceli, Gaetano Amato, Federico Martinelli

**Affiliations:** 1 Dipartimento di Scienze Agrarie e Forestali, Università degli Studi di Palermo, Palermo, Italy; 2 Fondazione A. e S. Lima Mancuso, Università degli Studi di Palermo, Palermo, Italy; Estación Experimental del Zaidín (CSIC), SPAIN

## Abstract

Arbuscular mycorrhizal fungi (AMF) have a major impact on plant nutrition, defence against pathogens, a plant’s reaction to stressful environments, soil fertility, and a plant’s relationship with other microorganisms. Such effects imply a broad reprogramming of the plant’s metabolic activity. However, little information is available regarding the role of AMF and their relation to other soil plant growth—promoting microorganisms in the plant metabolome, especially under realistic field conditions. In the present experiment, we evaluated the effects of inoculation with AMF, either alone or in combination with plant growth–promoting rhizobacteria (PGPR), on the metabolome and changes in metabolic pathways in the roots of durum wheat (*Triticum durum* Desf.) grown under N-limited agronomic conditions in a P-rich environment. These two treatments were compared to infection by the natural AMF population (NAT). Soil inoculation with AMF almost doubled wheat root colonization by AMF and decreased the root concentrations of most compounds in all metabolic pathways, especially amino acids (AA) and saturated fatty acids, whereas inoculation with AMF+PGPR increased the concentrations of such compounds compared to inoculation with AMF alone. Enrichment metabolomics analyses showed that AA metabolic pathways were mostly changed by the treatments, with reduced amination activity in roots most likely due to a shift from the biosynthesis of common AA to γ-amino butyric acid. The root metabolome differed between AMF and NAT but not AMF+PGPR and AMF or NAT. Because the PGPR used were potent mineralisers, and AMF can retain most nitrogen (N) taken as organic compounds for their own growth, it is likely that this result was due to an increased concentration of mineral N in soil inoculated with AMF+PGPR compared to AMF alone.

## Introduction

Most land plants live in symbiosis with AMF. This mutualistic symbiosis plays a crucial role in the uptake of nutrients by the host plant and its defence against pathogens and frequently increases plant growth and stress resistance [1,2]. From a nutritional perspective, AMF can dominate the uptake of phosphorus (P) and other nutrients with low mobility in soil even in the absence of any plant growth response [3]. Yet their role in the uptake of nitrogen (N) and its importance for plant N nutrition is still unclear [4] and can vary according to the nutrient status of the host plant [5,6]; the type of nutrient supplied to the soil [7,8] and its availability; and interactions among the plant, AMF, and soil bacteria [8–10]. In addition, the arbuscular mycorrhizal (AM) fungal partner can influence important plant transcriptomic pathways related to plant N uptake and metabolism [11–13] and other metabolic pathways [14]. Thus, AMF can influence a wide range of plant metabolic processes. Such effects can be due to the effects of AMF on resource availability, which has direct major implications for the regulation of metabolic networks [15–18]; to the presence of the AM fungal partner itself [19]; to variations in the energy cost of the uptake of nutrients and feeding of plant-associated microbes [20]; and to their interactions with the plant and other soil microbes [21,22]. Among the soil microorganisms different than AMF, plant growth promoting rhizobacteria (PGPR) are likely to influence both plant growth and plant-AMF relationships mainly through indirect mechanisms including an increase in soil nutrient availability [23] whereas their direct effect on plant growth are still under debate [24]. However, few studies have evaluated the effects of either AMF or other plant growth–promoting microorganisms on plant metabolic pathways, and most that have been conducted under controlled greenhouse conditions in sterilised uninoculated media as a control [24–26] or have analysed only target compounds [27,28]. Metabolomics has improved at elucidating complex metabolic pathways thanks to increasing standardization of extraction and analytic procedures, especially gas chromatography (GC)-electronic ionization (EI)-mass spectrometry (MS)–based techniques, and increasing compound annotation in databases [29–32]. The aim of the present experiment was to evaluate the effects of AMF field inoculation, alone or in combination with a consortium of PGPR efficient at mineralising organic matter, on the metabolome and changes in metabolic pathways in the roots of durum wheat (*Triticum durum* Desf.) grown under N-limited, P-rich field conditions. Such condition was chosen since it has been shown that N limitation can reduce the AM benefit for the plant, especially under high P availability [6]. We hypothesized that a competition occurs between AM fungi and plants for nitrogen coming from the soil organic matter and that this should rearrange the metabolome of the plant root. An additional hypothesis is that the ability of PGPR to release N from the native organic matter should consist in an increase of N availability and thus reduce the competition for N between plant and AM fungus.

## Materials and Methods

### Ethics Statement

No specific permits were required for the described field study. The location is not protected in any way. The experiment did not involve endangered or protected species.

### Experimental design

A field trial was performed in 2011–2012 in a typical semiarid Mediterranean area (37°33’ N–13°31’E, 178 m a.s.l.) on a deep, well-structured soil classified as a Vertic Xerochrept. Soil characteristics (0–0.60 m layer) were as follows: 52% clay, 25% sand, pH 8.2 (1:2.5 H_2_O), 16.8 g kg^−1^ total carbon (C; Walkley—Black), 1.78 g kg^−1^ total N (Kjeldahl), 92 mg kg^−1^ available P_2_O_5_ (Olsen), 1.37 g kg^−1^ total P_2_O_5_, 35 cmol kg^−1^ cation exchange capacity, 37.2% water content at field capacity, and 19.6% at the permanent wilting point. The climate at the experimental site is semiarid Mediterranean. From September to March, mean rainfall is 490 mm and mean air temperature ranges from 9.2°C to 11.9°C. During the cropping season, total rainfall (513 mm) was well distributed, whereas air temperature was 1.3°C lower than the long-term average. Weather data were collected from a weather station located within 500 m of the experimental site. Soil was cropped in the previous growing season with durum wheat. Before the experiment started, soil was ploughed at a depth of 30 cm in the summer and then shallow harrowed twice to control weeds and prepare suitable seedbed conditions. The natural AM spore population in the field measured before sowing by the wet-sieving method consisted of mainly by *Glomus*-group AM species and *Acaulospora* at an overall AM spore density of 5 spores per 100 g air-dried soil.

The experiment included three treatments (replicated six times): uninoculated control (NAT), inoculation with AMF alone (AMF), and inoculation with both AMF and PGPR (AMF+PGPR). Plots were arranged according to a randomised block design. Inoculation with AMF included the application of a commercial polispecies inoculum (Micronised Endo Mycorrhizae; Symbio, Wormley, Surrey, Great Britain) at a rate of 1.55 g m^−2^ at the time of sowing. The inoculum was composed of the following AM species: *Scutellospora calospora*, *Acaulospora laevis*, *Glomus aggregatum*, *Rhizophagus irregulare* (syn *G*. *intraradices*), *Funneliformis mosseae* (syn *G*. *mosseae*), *G*. *fasciculatum*, *G*. *etunicatum* e *G*. *deserticola*, and *Gigaspora margarita*. The inoculum was composed of 95% AM spores and 5% organic material. Total spore density in the inoculum was 25 g^−1^ per species. Inoculation with AMF+PGPR was performed by applying to the soil both 1.55 g m^−2^ AM inoculum as previously described and 1.55 g m^−2^ of a commercial PGPR inoculum. The PGPR inoculum was also purchased from Symbio (*Bacillus* Sp. on bran; Symbio, Wormley, Surrey, Great Britain) and was composed of *Bacillus amyloliquefaciens*, *B*. *brevis*, *B*. *circulans*, *B*. *coagulans*, *B*. *firmus*, *B*. *halodenitrificans*, *B*. *laterosporus*, *B*. *licheniformis*, *B*. *megaterium*, *B*. *mycoides*, *B*. *pasteurii*, *B*. *polymyxa*, and *B*. *subtilis*, each at a density of 2 billion cfu g^−1^. Durum wheat (cv. Anco Marzio, 1000-seed weight 47.4 g, 95% germination) was sown on 16 December 2012 at a rate of 350 seeds m^−2^ in rows 18.75 cm apart. The experimental plot consisted of eight rows, each 6 m long. Each experimental plot was spaced 0.5 m out from the next to avoid cross inoculation among treatments and 0.5-m wide corridors were tilled once per month to avoid AMF and PGPR movements across plots. Weeds were controlled by hand during the experiment. At wheat tillering (110 days after sowing), the aboveground biomass of a subplot (six rows 75 cm long) was harvested and weighed and a subsample of 1 kg fresh matter was taken and oven dried at 70°C until a constant weight. Dry mass was determined and further analysed for total N (Kjieldhal) and P (Bertramson), the latter after 48 h of heating at 550°C and no addition of magnesium nitrate. Roots (0–0.30 m layer) from five random plants from each plot were also sampled and two root subsamples of about 3 g were taken. The first subsample was immediately freeze dried in liquid N to stop metabolic activity and stored at—80°C for further analysis. The other subsample was stained with 0.05% trypan blue in lactic acid according to [33] and root colonization by AM fungi was measured using the grid intersect method as described in [34].

### Metabolite extraction and derivatization

Roots were lyophilised and ground in liquid N, and a 20-mg aliquot was processed as follows according to [35,36]. Samples were added to 0.75 ml methanol:chloroform:water (5:2:2), agitated at 4°C for 5 min, vortexed briefly, and then centrifuged at 6000 rpm for 2 min. After centrifugation, 0.60 ml supernatant was collected and dried in a SpeedVac. The dried extract was added to 2 ml of an internal retention index (from C8 to C16 at 0.8 mg/ml and from C18 to C30 at 0.4 mg/ml) and 5 μl of a methoxyamine hydrochloride solution (20 mg/ml in pyridine) and then shaken at 30°C for 90 min. Then 45 μl N-Methyl-N-(trimethylsilyl) trifluoroacetamide (MSTFA) with 1% trimethylchlorosilane (TMCS) for trimethylsilylation was added and the solution was shaken at 37°C for 30 min.

### GC-TOF-MS analysis and spectra processing

GC-MS analysis was performed on an Agilent 6890 gas chromatograph coupled with a Leco Pegasus III TOF mass spectrometer controlled by Leco ChromaTOF software 2.32. The gas chromatograph was equipped with an Rtx-5Sil MS column (Restek, Bellefonte, PA; 30 m long, 0.25 mm i.d. with 0.25 μm 95%-dimethyl-5% diphenyl polysiloxane film) and an additional 10 m integrated guard column. GC-time of flight (TOF)-MS analysis was performed according to [37]. Briefly, helium (99.99% purity) was used as a carrier gas at a constant flow of 1 ml/min. Oven temperature was held at 50°C for 1 min, then increased 10°C/min to 330°C, after which it was held at 330°C for 5 min. The transfer line was held at 280°C and the ion source at 250°C. Ionization was performed at 70 eV. A 290-s solvent delay was set. The mass range was set at 85–600 mass units acquired at 10 spectra s^−1^ and 1800 V detector voltage with no mass defect option. Resolution was 1 ppm with 2% error for isotopic abundance patterns. Spectra were compared to those present in the Fiehn Library (fiehnlab.ucdavis.edu/projects/FiehnLib, including 1,013 metabolites on 2014) and NIST 2005 with at least 95% matching.

### HILIC-Q-TOF-MS analysis and spectra processing

HILIC-Q-TOFMS analysis (hydrophilic interaction chromatography time of flight mass spectrometry) was performed on an Agilent 1290 UHPLC equipped with a Waters Acuity 1.7 μm BEH HILIC 2.1 × 150 mm column for separation and coupled with an Agilent G6530A accurate-mass QTOF equipped with an Agilent ESI Jet Stream ion source. Mobile phases were Solvent A (5mM ammonium acetate with 0.2% acetic acid) and Solvent B (9:1 acetonitrile:water with 5mM ammonium acetate and 0.2% acetic acid). Dry samples were resuspended in 100 μl solvent B and then injected in the column under the following gradient conditions: from 0 to 4 min, isocratic 100% B; from 4 to 12 min, B linearly reduced to 45%; from 12 to 20 min, isocratic 45% B. After the run, there was a 20-min re-equilibration phase before the next sample was injected. The following Source/MS conditions were applied: electrospray ionization (ESI) set in positive mode, 3000 V capillary voltage; source gas temperature set to 350°C and gas flow to 10 L/min, mass range acquisition 50–1700 Da at 4 scans per second. After deconvolution, raw data were aligned and adducts [M+H^+^] searched against METLIN, NIST MS, and MZmine software. Metabolite intensities were identified as the peak height for the largest precursor ion (not normalized).

### Annotation and statistical analyses

One way analysis of variance (ANOVA) [38] was performed for biomass, N and P content and uptake, and percent root colonization by AMF. When treatments were significant, least significant differences were computed to separate means. Enrichment metabolomics analyses were performed on *Zea mays* precompiled background sets in MBRole [39], and analyses of metabolic pathways were performed using Pathos [40] with minimum variation set at 20%. The *P* value of annotation for Biological Role in MBRole was adjusted for multiple testing using the false discovery rate according to [41].

All data for annotated and unannotated identified GC and LC peaks were standardised by setting the mean to 0 and the standard deviation to 1. This allowed the computation of means of different compounds with the same biological role without algebraic distortions due to a different actual concentration—to—MS signal ratio. Biological role means of standardised data from annotated and unannotated identified GC peaks were computed and ANOVAs were run on these means to highlight average variations in compounds in the same biological group. Canonical discriminant analysis (CDA) [38] was run using biological group means as vectors to summarize between-treatments variation. The percentage of compounds not varying or varying at either >+20% or <−20% of the base condition was drawn from Pathos results for each metabolic pathway significantly annotated in MBRole.

## Results

### Plant growth

**Fig 1:**
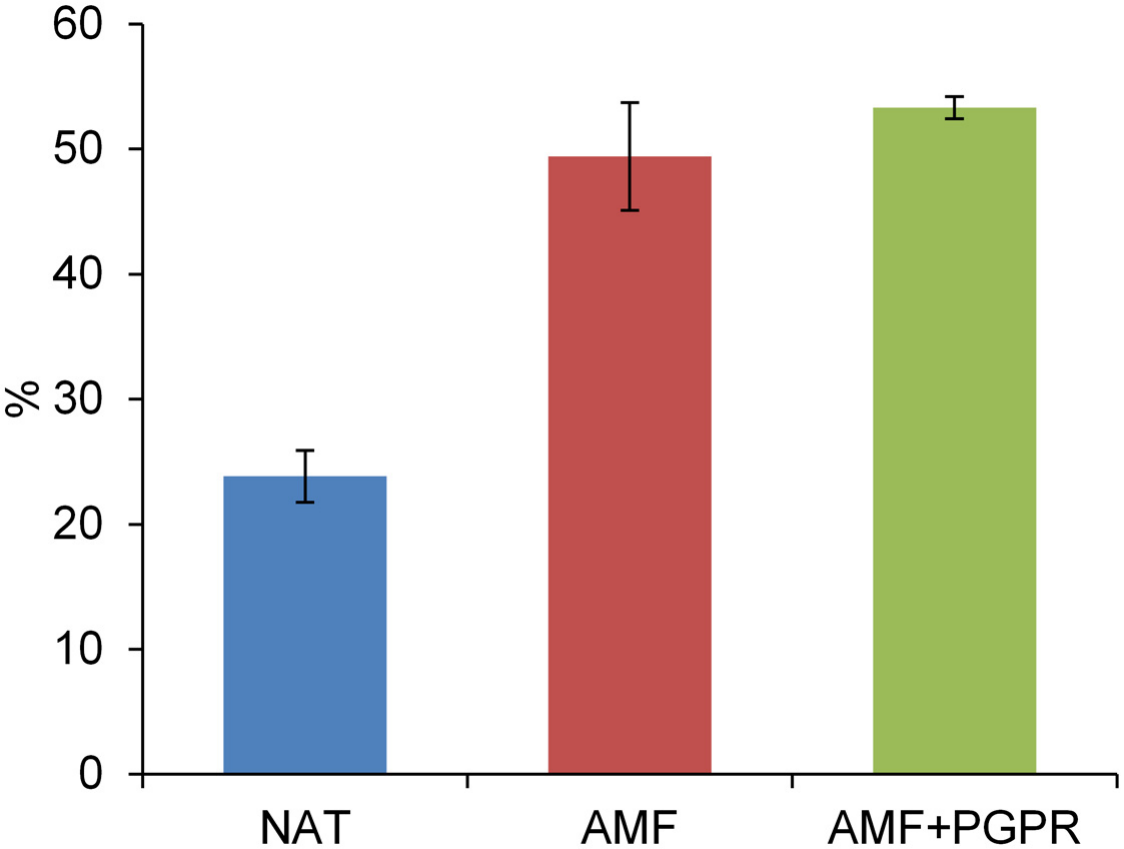
Root colonization by AMF in roots of durum wheat. Wheat with natural arbuscular mycorrhizal inoculum (NAT), inoculated with AM fungi spores (AMF), or inoculated with both AMF and plant growth—promoting rhizobacteria (AMF+PGPR). Data are means±S.E. (n = 6).

Inoculation with AMF (either alone or in combination with PGPR) increased by 23.3% the aboveground biomass of durum wheat, although this change was not significant (Table 1). It also slightly decreased both N (−7.8%) and P (−8.0%) content. Soil inoculation with AMF almost doubled wheat root colonization by AM fungi (Fig 1). No effects of treatment on wheat above ground biomass, grain yield and yield components were observed at maturity (Table A in S1 File).

**Table 1:** Aboveground biomass, its N and P and root infection by AM fungi at tillering of durum wheat grown in the field.

	Aboveground biomass	N content	N uptake	P content	P uptake
	Mg ha^–1^	mg N g^–1^ biomass	kg N ha^–1^	mg P g^–1^ biomass	kg P ha^–1^
NAT	1.52±0.177	18.78±0.970	28.39±2.441	3.60±0.117	5.40±0.260
AMF	1.85±0.240	16.88±0.383	31.30±4.176	3.37±0.173	6.17±0.733
AMF+PGPR	1.89±0.223	17.78±0.868	34.25±5.574	3.25±0.075	6.17±0.793
*P-value*	*0*.*106*	*0*.*236*	*0*.*253*	*0*.*153*	*0*.*525*

NAT = wheat with natural arbuscular mycorrhizal inoculum; AMF = wheat inoculated with spores of AM fungi; AMF+PGPR = wheat inoculated with both spores of AM fungi and plant growth—promoting rhizobacteria.

### Biological classes

**Fig 2:**
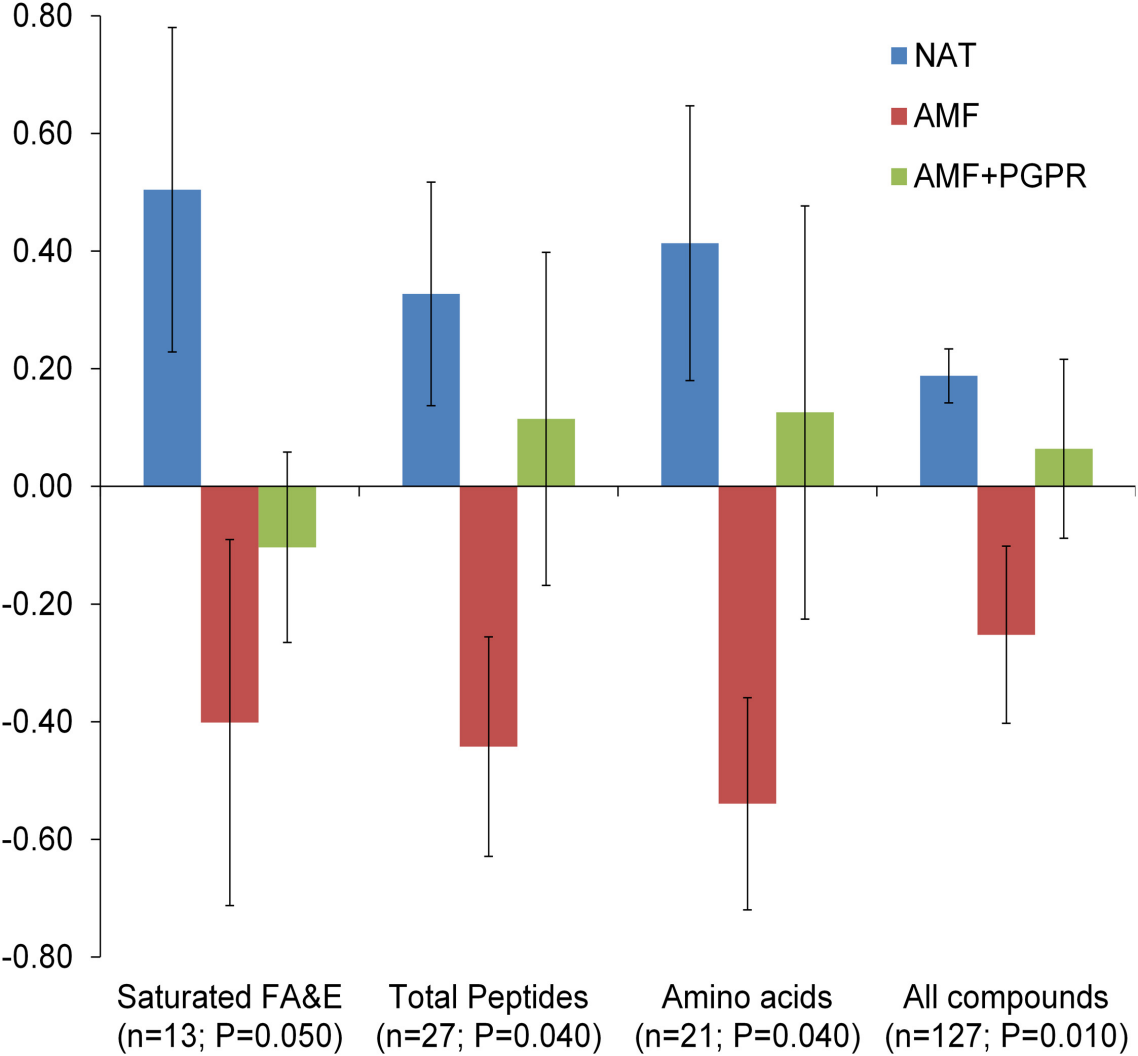
Group means ± S.E. across standardised data for identified GC peaks grouped per biological group significantly varying according to the treatments. n indicates the number of compounds contributing to the relative mean, and P is the *P* value of the ANOVA for that group. Carbohydrates were analysed separately according to KEGG annotation. All compounds includes both annotated and unannotated compounds. GC was run with methanol:chloroform:water (5:2:2) extracts from roots of durum wheat grown in the field with natural arbuscular mycorrhizal inoculum (NAT), inoculation with AM fungi (AMF), or inoculation with both AMF and plant growth—promoting rhizobacteria (PGPR). FA&E, fatty acids and their esters. Please see Table G in S1 File for non-significantly varying classes of compounds.

**Fig 3:**
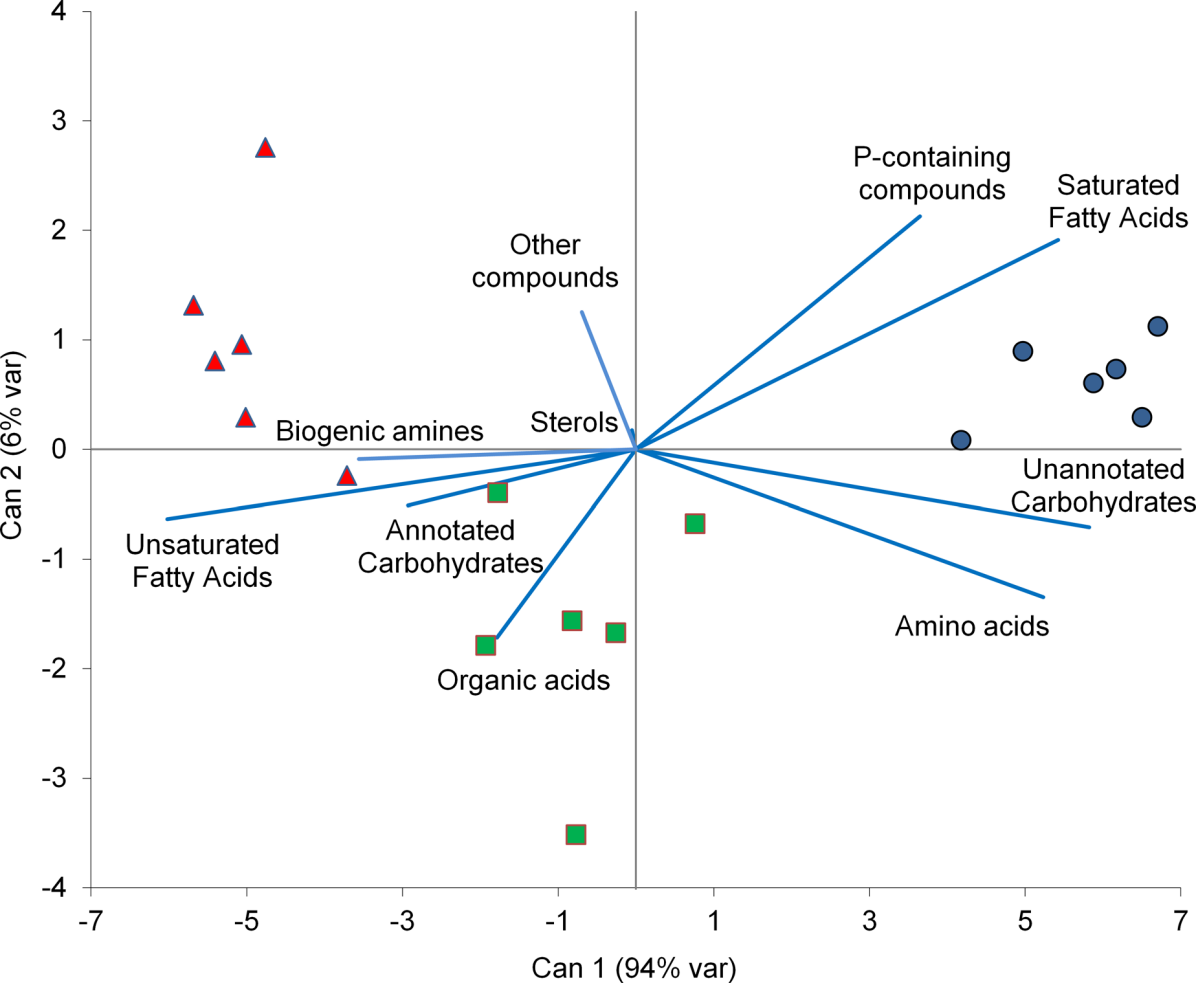
CDA run using biological group means of standardised data from identified GC peaks as vectors. The percentage of the total variance explained by each canonical axis is shown in parentheses. NAT, blue circles; AMF, red triangles; AMF+PGPR, green squares. Please note that CDA vectors do not represent perpendicular directions through the space of the original variables. Fatty acids vectors include both fatty acids and their esters.

A total of 315 GC peaks were found, 118 of which clearly identified and 9 of which were assigned to a carbohydrate chemical structure. A Kegg ID was given to only 83 compounds and used for an enrichment metabolomics analysis against *Z*. *mays* precompiled background sets in MBRole, which annotated only 73, 38, 44, 64, and 28 Kegg IDs in Pathways, Enzyme interactions, Biological role, Chemical groups, and Other interactions, respectively (Tables A, B, C, D, and F in S1 File, respectively). In general, AMF showed a lower concentration of most GC-separated compounds than NAT, especially saturated fatty acids (SaFA) and AA (Fig 2 and Table G in S1 File). No differences were observed in the concentrations of any compounds between AMF+PGPR and either NAT or AMF (Fig 2). CDA run using classes of compounds from GC only separated AMF from NAT (Fig 3). Canonical Variable 1 accounted for 94% of the total variance and mostly depended on unsaturated fatty acids (UnFA) (standardized canonical coefficient, SCORE = −6.02), unannotated carbohydrates (SCORE = +5.82), SaFA (SCORE = +5.42) and AA (SCORE = +5.23). Few correlations were found between Canonical Variable 2 and the concentrations of different classes of compounds included in the CDA. When root extract was separated by HILIC, 516 peaks were found and 52 compounds were assigned a simple empirical formula (Table H in S1 File). Among them, 80% contained P in their structures. HILIC-separated compounds included carnitines, glycerophosphocholine, lysophosphatidylcholines, phosphatidylcholines, lysophosphatidylethanolamines, and phosphatidylethanolamines. In particular, lysophosphatidylcholine 16:0 was lower in AMF than NAT, whereas concentrations of the other compounds, both singly or grouped by chemical class, did not vary significantly by treatment. CDA run using HILIC-separated compounds did not discriminate among treatments (data not shown).

### Metabolic pathways

**Fig 4:**
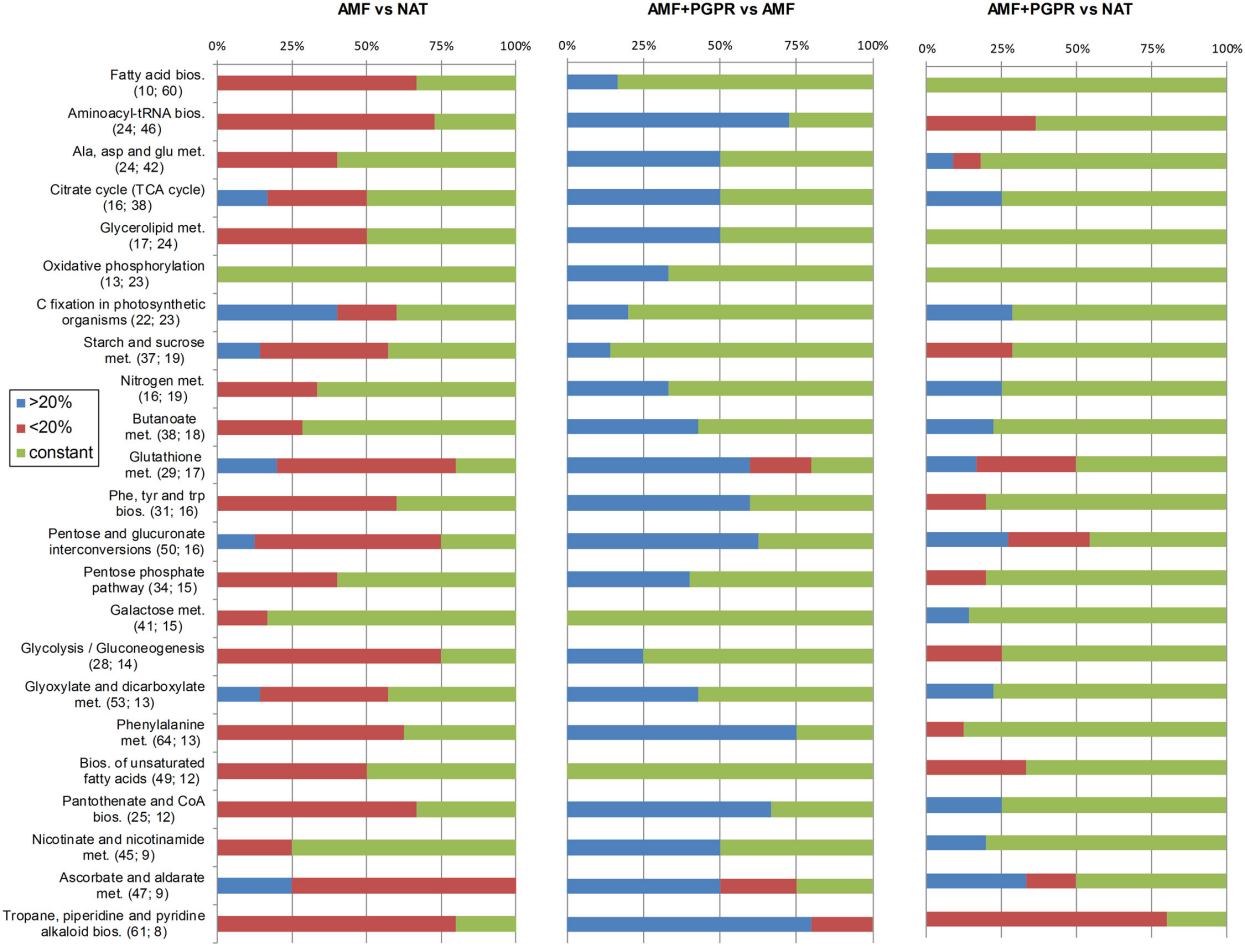
The percentage of annotated compounds included in Pathos in each metabolic pathway. Compounds are displayed as unchanged (green bars), or significantly increased (blue bars), or significantly decreased (red bars) at 20% minimum variation. The first number in parentheses indicates the total number of annotated compounds in the pathway, and the second number indicates the percentage of annotated compounds from the present data set in each metabolic pathway. Only those pathways that were significantly annotated in MBRole are shown.

A total of 25 metabolic pathways were significantly annotated on *Z*. *mays* background in MBRole, and 23 of these were analysed in Pathos on *Arabidopsis thaliana* background. Symbiosis with AM fungi reduced the concentrations of most compounds in all metabolic pathways. No changes were observed for oxidative phosphorylation. AMF mostly upregulated metabolites involved in carbon fixation in photosynthetic organisms (Fig 4). AMF+PGPR mostly increased the concentrations of compounds in all metabolic pathways except for galactose metabolism and the biosynthesis of unsaturated fatty acids.

Metabolic enrichment analysis showed that AMF down-regulated key pathways belonging to the backbone of primary metabolism, such as amino acid biosynthesis and interconversions, especially those involving alanine, glutamine, asparagine, and phenylalanine (Table C in S1 File); TCA cycle and glycolysis; and carbohydrate biosynthesis and metabolism (sugar, starch, pentose phosphate, galactose; Table B in S1 File). Lipid-related pathways, such as fatty acid biosynthesis and glicerolipid metabolism, were also down-regulated.

Peptide metabolic pathways were more depressed in AMF and AMF+PGPR compared to NAT. In particular, a reduction in products formed by asparagine synthase (glutamine-hydrolysing; EC 6.3.5.4) and glutamate-ammonia ligase (EC 6.3.1.2) was observed in different metabolic pathways, such as alanine, aspartate, and glutamate metabolism (map 00250) and arginine and proline metabolism (map 00330). In addition, a lower abundance of compounds included in the urea cycle (map 00330), especially ornithine and its direct product, putrescine (data not shown), was found in AMF than NAT. Gamma-amino butyric acid (GABA) was slightly higher in AMF than NAT. Only one metabolic pathway leading to GABA formation was higher in AMF than NAT (glutamate decarboxylase, EC 4.1.1.15). Because all other GABA-forming pathways showed reduced concentrations of the other metabolites directly leading to GABA (namely, succinic acid in butanoate metabolism [map 00650] and putrescine [map 00330]) and an increase in some precursors of these metabolites (maleate in map 00650), it is likely that AMF reduced amination activity in roots compared to NAT. Compared to AMF, AMF+PGPR reduced both GABA and one of its direct precursors, glutamic acid, and increased other GABA precursors, the compounds in the urea cycle, and glutamine, which is formed from glutamic acid in competition with GABA formation. Finally, AMF+PGPR reduced the concentration of pipecolic acid compared to either AMF or NAT, although no differences were observed between AMF and NAT.

AMF increased the concentration of xilitol and reduced its derivatives (D-xilose, D-arabitol, and L-arabinose) in pentose and the glucuronate interconversions pathway (map 00040) compared to NAT. This led to a reduction in 2-oxoglutarate and pyruvate. In contrast, AMF+PGPR increased D-arabitol, 2-oxoglutarate, pyruvate, and L-arabinose compared to AMF but not NAT. D-xilose (map 00040) can also derive from the metabolism of starch and sucrose (map 00050), where few compounds were annotated (7 out of 37), and none of these were directly related to D-xilose. This allows for little elucidation of the latter pathway. Finally, AMF and AMF+PGPR increased the concentration of malic acid compared to NAT in carbon fixation in photosynthetic organisms (map 00710). On the one hand, malic acid is directly converted to pyruvate by means of a malate dehydrogenase (EC 1.1.1.40) under light conditions in map 00710. On the other hand, pyruvate can undergo different transformations in pyruvate metabolism (map 00620): it can be indirectly converted to malic acid to feed the glycolysis/glucogenesis, glyoxylate and citrate cycles; it can be indirectly or directly interconverted with lactic acid by means of an L-lactate dehydrogenase (EC 1.1.1.27); or it can be transferred to the biosynthesis of leucine, lysine, or fatty acid or the metabolism of propanoate, butanoate, and ketone bodies. We found that pyruvate decreased in AMF compared to NAT and increased in AMF+PGPR compared to AMF, whereas lactic acid was lower in AMF and AMF+PGPR than in NAT. Finally, annotation indicated that two out of 4 sterols (namely zymosterol, Kegg: C05437; and ergosterol, Kegg: C01694) were indicated as potentially deriving from the fungi. However, no differences in zymosterol concentration among treatments were found, and ergosterol was 36.5% and 41.5% lower in AMF and AMF+PGPR, respectively, if comparing to NAT, thus we exclude that these sterols were deriving from the AMF. In general, steroid biosynthesis (map 00100, adjusted *P* annotation = 0.054 in MBRole) was slightly depressed by AMF and AMF+PGPR compared to NAT. No metabolite potentially belonging to bacteria was found.

## Discussion

Above ground plant N content was on average 17.81 mg N g^−1^ dry matter. Such N content at tillering can be considered as very low [42]. This indicates that the N availability in the soil was limiting plant growth, as observed by [43]. This was likely because of the intrinsic low soil N content and the previous cultivation of wheat, an N-depleting species.

Root AM infection of plots with natural AM inoculum (NAT) was on average slightly higher than 20%, which is similar to [44] but markedly lower than [45,46]. This suggests that, in our experiment, the actual soil conditions were unfavourable for the infection of roots by the natural AM consortium, probably because of several factors, such as the high intensity of the tillage technique adopted (mouldboard ploughing) and the high availability of phosphorus in the soil, both of which are detrimental to natural AM infection [45,47,48]. Soil inoculation with AM fungi, either alone or in combination with PGPR, markedly increased wheat root colonization by AM fungi, as in other studies [44,48].

Means across standardised data of identified compounds suggested that soil inoculation with AMF alone decreased concentrations of low molecular weight compounds in roots, particularly AA and saturated FA&E. Similar results were found by [49] for *Lotus japonicus* grown with or without AM fungi under different conditions. Information about the effects of AMF on the root metabolome is scarce. Other experiments showed that in the legume *Medicago truncatula*, AMF increased the concentration of AA in the roots [19]. In *Lolium perenne*, colonisation by *Neotyphodium lolii*, a fungal endophyte, reduced the content of nitrate and several amino acids in the host plant [50]. Hodge and Fitter [51] showed that AM fungi have a high N demand and retain most N taken as organic compounds for their own growth. In the present experiment, native organic matter and wheat roots of the previous crop were the only sources of N available for plants. Thus, it is possible that the greater development of AM hyphae in the soil in AMF than NAT resulted in a reduced N available to the plant. Thus, the reduced amination activity in AMF than NAT observed in the present study could have resulted either directly from increased root mycorrhization, as shown in *L*. *japonicus* [49], or indirectly from reduced N availability for the plant in AMF than NAT. This is consistent with the finding that GABA, which is synthesised in competition with many other AA, increased in AMF compared to NAT, as this molecule is implicated in a wide range of plant responses to external factors, including plant microbe interaction and biotic and abiotic stresses [52] and nutrient limitation [53]. This compound is an important constituent of root exudates that alters the growth and activity of some PGPR [54–56]. Accordingly, the higher amino acid content in AMF+PGPR than AMF may be due to the mineralisation activity by PGPR and to the further increase in N availability, especially ammonium and nitrate for plants. Indeed, the *Bacillus* species used in the present study are sturdy plant growth promoters that could have benefitted plants through their mineralisation activity or by reducing competition with other bacteria. This is corroborated by the reduced concentration of pipecolic acid in AMF+PGPR compared to AMF or NAT. Pipecolic acid is implied in systemic acquired resistance against bacteria and stresses [57–59] and accumulates into the plant at increasing N availability [60]. *Bacillus* volatiles, especially 2,3-butanediol and acetoin, promote plant growth [61]. However, in the present study, the only annotated compound implicated in the 2,3-butanediol/acetoin/GABA system (map 00650) was pyruvate, which is included in 25 different annotated metabolic pathways. This should curb any speculation about any effect of PGPR on wheat metabolic reprogramming. Fatty acids and their esters and carbohydrates, especially those that are unannotated, also contributed to separate AMF samples from NAT but not from AMF+PGPR. Simple sugars are the most important carbon sources exported from roots to the intra-radical mycelium (IRM) [62]. Just as lipids are synthesised into the IRM, these sugars consistently flow to the the extra-radical mycelium (ERM) [63]. AM symbiosis and N and P availability can affect the fatty acid content in roots [64]. However, lipids arising from AM fungi usually emerge in the roots in the late stage of symbiosis [65]. This could explain why we observed no variation in carnitines, which accumulate in mycorrhizal roots only in the late stage of AM symbiosis [66]. In early, active stages, high demand for lipids by ERM [67] can result in a reduction of lipids in mycorrhizal roots compared to non-mycorrhizal roots.

## Conclusions

The present data, obtained through metabolomics analyses of field grown wheat, showed that inoculation with AMF negatively affected amination activity in the root and concentrations of most of amino acids. Inoculation with AMF+PGPR increased concentrations of amino acids compared to AMF. This support the hypothesis that N availability is crucial for the AM benefit to the plant and that AM fungi can compete with the plant for N coming from the organic matter. Because most N taken directly in organic form from AM fungi is retained in AM structures [51], this result is likely due to the increase in soil inorganic N in AMF+PGPR compared to AMF. Inoculation of the soil with exotic AMF also resulted in a reprogramming of primary metabolism, with a clear shift from the biosynthesis of common AA to GABA. The low percentage of compound annotation in plant metabolic pathways (ca. 23% of GC peaks) obscures the metabolic reprogramming at play in carbohydrate metabolism. Nonetheless, many compounds implicated in C fixation were increased in AM rather than non-AM roots, and this agrees with the findings of several authors reporting increased C fixation even in the absence of a significant growth response [19,28,64]. In addition, the increase in xilitol, which is crucial in the interaction between plants and AM fungi [19,49,68], suggests that plants were actively feeding the fungal symbionts.

## Supporting Information

S1 File Supporting information file. Above ground biomass, grain yield and yield components at maturity of durum wheat grown in the field (**Table A**). Enrichment metabolomic analysis for Pathways (**Table B**), Enzyme interactions (**Table C**), Biological role (**Table D**), Chemical groups (**Table E**), and Other interactions (**Table F**) as displayed in MBRole. Group means ± S.E. across standardised data for identified GC peaks grouped per biological group (**Table G**). HILIC-Q-TOF MS identified compounds (**Table H**).
(DOCX)
