# Supplementary material for: Metabolomics Suggests That Soil Inoculation with Arbuscular Mycorrhizal Fungi Decreased Free Amino Acid Content in Roots of Durum Wheat Grown under N-Limited, P-Rich Field Conditions
Source: PLoS One. 2015 Jun 11;10(6):e0129591. doi: 10.1371/journal.pone.0129591 (PMC4466249; doi:10.1371/journal.pone.0129591)
Supplement: S1 File — Above ground biomass, grain yield and yield components at maturity of durum wheat grown in the field (Table A). Enrichment metabolomic analysis for Pathways (Table B), Enzyme interactions (Table C), Biological role (Table D), Chemical groups (Table E), and Other interactions (Table F) as displayed in MBRole. Group means ± S.E. across standardised data for identified GC peaks grouped per biological group (Table G). HILIC-Q-TOF MS identified compounds (Table H). (DOCX) [file pone.0129591.s001.docx]

**Table A.** Above ground biomass, grain yield and yield components at maturity of durum wheat grown in the field. Wheat with natural arbuscular mycorrhizal inoculum (NAT), inoculated with AM fungi spores (AMF), or inoculated with both AMF and plant growth–promoting rhizobacteria (AMF+PGPR). Yield components are number of spikes per square meter, number of kernels per spike, and 1000-kernel weight.

|  | Above ground biomass | | | Grain yield | | | Spikes per m^–2^ | | | Kernels  per spike | | | 1000-kernel weight | | |
| --- | --- | --- | --- | --- | --- | --- | --- | --- | --- | --- | --- | --- | --- | --- | --- |
|  | Mg ha^–1^ | | | Mg ha^–1^ | | |  | | |  | | | g | | |
| NAT | 9.0 | ± | 0.21 | 3.8 | ± | 0.12 | 255 | ± | 9.8 | 31.5 | ± | 1.72 | 47.1 | ± | 0.39 |
| AMF | 9.2 | ± | 0.25 | 3.8 | ± | 0.15 | 266 | ± | 13.0 | 32.4 | ± | 3.83 | 45.7 | ± | 3.25 |
| AMF+PGPR | 9.0 | ± | 0.25 | 3.7 | ± | 0.09 | 252 | ± | 12.4 | 31.3 | ± | 1.84 | 47.3 | ± | 0.98 |
| *P-value* | *0.806* | | | *0.834* | | | *0.673* | | | *0.935* | | | *0.837* | | |

**Table B.** Enrichment metabolomic analysis for Pathways as displayed in MBRole.

| **Pathways (set: 73 background: 3327)** | ***P*-value** | **Adjusted *P*-value*** | **In background** | **In set** | **%**** | **Compounds** |
| --- | --- | --- | --- | --- | --- | --- |
| Alanine aspartate and glutamate metab. | 0.000 | 0.000 | 24 | 10 | 13.7 | C00334 C00022 C00122 C00064 C00041 C00026 C00152 C00025 C00042 C00049 |
| Aminoacyl-tRNA bios. | 0.000 | 0.000 | 75 | 11 | 15.1 | C00047 C00079 C00082 C00152 C00188 C00041 C00049 C00078 C00064 C00407 C00025 |
| Citrate cycle (TCA cycle) | 0.000 | 0.000 | 20 | 6 | 8.2 | C00122 C00149 C00026 C00022 C00158 C00042 |
| Metabolic pathways | 0.000 | 0.000 | 1455 | 51 | 69.9 | C00198 C00026 C00064 C00116 C06427 C00025 C00085 C00811 C00156 C00099 C05437 C00158 C00134 C00077 C00259 C00122 C00042 C05422 C00093 C00188 C00009 C00095 C00208 C00257 C01595 C00212 C00047 C00089 C00249 C00041 C00152 C00180 C00049 C00379 C00189 C00092 C00181 C01694 C00387 C00031 C00334 C00474 C00149 C00078 C00407 C00186 C00022 C00408 C00079 C00082 C00493 |
| Phenylalanine metab. | 0.000 | 0.000 | 46 | 8 | 11 | C00082 C00022 C00811 C00042 C00156 C00180 C00122 C00079 |
| Butanoate metab. | 0.000 | 0.000 | 40 | 7 | 9.6 | C00025 C00042 C00026 C00022 C01384 C00334 C00122 |
| Fatty acid bios. | 0.000 | 0.001 | 49 | 7 | 9.6 | C00712 C00249 C06424 C08362 C01530 C01571 C02679 |
| Starch and sucrose metab. | 0.000 | 0.001 | 50 | 7 | 9.6 | C00089 C00185 C00095 C00208 C00181 C00092 C00031 |
| Carbon fixation in photosynthetic organisms | 0.000 | 0.001 | 23 | 5 | 6.8 | C00049 C00041 C00149 C00022 C00085 |
| Pentose and glucuronate interconversions | 0.000 | 0.001 | 53 | 7 | 9.6 | C00085 C00181 C00379 C00474 C00259 C00022 C01904 |
| Nitrogen metab. | 0.000 | 0.001 | 26 | 5 | 6.8 | C00049 C00064 C00152 C00025 C00192 |
| Galactose metab. | 0.000 | 0.001 | 41 | 6 | 8.2 | C00089 C01235 C00031 C05401 C00095 C00116 |
| Phenylalanine tyrosine and tryptophan bios. | 0.000 | 0.001 | 27 | 5 | 6.8 | C00078 C00296 C00493 C00082 C00079 |
| Arginine and proline metab. | 0.000 | 0.002 | 82 | 8 | 11 | C00025 C00077 C00134 C00122 C00022 C00334 C00064 C00049 |
| Pentose phosphate pathway | 0.001 | 0.002 | 32 | 5 | 6.8 | C00022 C00198 C00257 C00031 C00121 |
| Bios. of unsaturated fatty acids | 0.001 | 0.004 | 54 | 6 | 8.2 | C06427 C01595 C00712 C00249 C06425 C01530 |
| Glutathione metab. | 0.001 | 0.005 | 38 | 5 | 6.8 | C00025 C01879 C00077 C05422 C00134 |
| Glyoxylate and dicarboxylate metab. | 0.002 | 0.009 | 44 | 5 | 6.8 | C00042 C00022 C00026 C00149 C00158 |
| Glycolysis / Gluconeogenesis | 0.004 | 0.014 | 31 | 4 | 5.5 | C01451 C00022 C00186 C00031 |
| Glycerolipid metab. | 0.005 | 0.014 | 32 | 4 | 5.5 | C00093 C00116 C02457 C05401 |
| Oxidative phosphorylation | 0.005 | 0.014 | 16 | 3 | 4.1 | C00009 C00122 C00042 |
| Nicotinate and nicotinamide metab. | 0.015 | 0.042 | 44 | 4 | 5.5 | C00022 C01384 C00122 C00049 |
| Tropane piperidine and pyridine alkaloid bios. | 0.016 | 0.043 | 68 | 5 | 6.8 | C00408 C00407 C00134 C00047 C00079 |
| Ascorbate and aldarate metab. | 0.019 | 0.048 | 47 | 4 | 5.5 | C00022 C00026 C00259 C05422 |
| Pantothenate and CoA bios. | 0.020 | 0.050 | 27 | 3 | 4.1 | C00049 C00022 C00099 |
| Glycine serine and threonine metab. | 0.021 | 0.051 | 49 | 4 | 5.5 | C00049 C00188 C00078 C00022 |
| Valine leucine and isoleucine bios. | 0.022 | 0.051 | 28 | 3 | 4.1 | C00022 C00188 C00407 |
| Steroid bios. | 0.025 | 0.054 | 51 | 4 | 5.5 | C01753 C05437 C05442 C01694 |
| beta-Alanine metab. | 0.029 | 0.062 | 31 | 3 | 4.1 | C00099 C00049 C00334 |
| C5-Branched dibasic acid metab. | 0.032 | 0.062 | 32 | 3 | 4.1 | C00022 C00026 C00025 |
| Lysine bios. | 0.032 | 0.062 | 32 | 3 | 4.1 | C00026 C00047 C00049 |
| Pyruvate metab. | 0.032 | 0.062 | 32 | 3 | 4.1 | C00149 C00022 C00186 |
| Cysteine and methionine metab. | 0.033 | 0.062 | 56 | 4 | 5.5 | C00049 C00022 C02989 C00041 |
| Propanoate metab. | 0.043 | 0.079 | 36 | 3 | 4.1 | C00099 C00042 C00186 |
| Cyanoamino acid metab. | 0.060 | 0.106 | 41 | 3 | 4.1 | C00082 C00152 C00049 |
| Histidine metab. | 0.071 | 0.119 | 44 | 3 | 4.1 | C00025 C00026 C00049 |
| Taurine and hypotaurine metab. | 0.070 | 0.119 | 20 | 2 | 2.7 | C00041 C00022 |
| Bios. of secondary metabolites | 0.074 | 0.120 | 1038 | 29 | 39.7 | C00047 C00122 C00180 C00082 C00134 C00025 C00493 C00149 C00156 C00257 C00408 C00811 C00407 C00026 C00092 C01753 C00042 C00049 C00158 C00079 C00198 C00186 C05442 C00031 C00078 C00077 C00022 C00152 C00188 |
| Tyrosine metab. | 0.084 | 0.133 | 76 | 4 | 5.5 | C00122 C00022 C00082 C00042 |
| Thiamine metab. | 0.110 | 0.171 | 26 | 2 | 2.7 | C00022 C00082 |
| Phenylpropanoid bios. | 0.119 | 0.179 | 55 | 3 | 4.1 | C00082 C00079 C00811 |
| Vitamin B6 metab. | 0.155 | 0.229 | 32 | 2 | 2.7 | C00022 C00026 |
| Ubiquinone and other terpenoid-quinone bios. | 0.232 | 0.335 | 76 | 3 | 4.1 | C00082 C00811 C00156 |
| Glycerophospholipid metab. | 0.268 | 0.377 | 46 | 2 | 2.7 | C00093 C00189 |
| Lysine degradation | 0.276 | 0.380 | 47 | 2 | 2.7 | C00047 C00408 |
| Amino sugar and nucleotide sugar metab. | 0.298 | 0.402 | 87 | 3 | 4.1 | C00181 C00259 C00031 |
| Purine metab. | 0.328 | 0.433 | 92 | 3 | 4.1 | C00212 C00064 C00387 |
| Pyrimidine metab. | 0.373 | 0.482 | 59 | 2 | 2.7 | C00064 C00099 |
| Tryptophan metab. | 0.536 | 0.678 | 81 | 2 | 2.7 | C05659 C00078 |
| Porphyrin and chlorophyll metab. | 0.772 | 0.957 | 126 | 2 | 2.7 | C00188 C00025 |
| alpha-Linolenic acid metab. | 1.000 | 1.000 | 40 | 1 | 1.4 | C06427 |
| Biotin metab. | 1.000 | 1.000 | 11 | 1 | 1.4 | C00047 |
| Fatty acid metab. | 1.000 | 1.000 | 50 | 1 | 1.4 | C00249 |
| Fructose and mannose metab. | 1.000 | 1.000 | 48 | 1 | 1.4 | C00095 |
| Inositol phosphate metab. | 1.000 | 1.000 | 39 | 1 | 1.4 | C00092 |
| Isoquinoline alkaloid bios. | 1.000 | 1.000 | 94 | 1 | 1.4 | C00082 |
| Methane metab. | 1.000 | 1.000 | 34 | 1 | 1.4 | C00085 |
| Photosynthesis | 1.000 | 1.000 | 11 | 1 | 1.4 | C00009 |
| Riboflavin metab. | 1.000 | 1.000 | 21 | 1 | 1.4 | C00474 |
| Selenoamino acid metab. | 1.000 | 1.000 | 30 | 1 | 1.4 | C00041 |
| Terpenoid backbone bios. | 1.000 | 1.000 | 33 | 1 | 1.4 | C00022 |
| Valine leucine and isoleucine degradation | 1.000 | 1.000 | 41 | 1 | 1.4 | C00407 |
| Compounds with no annotations (10): C08240 C01389 C01601 C00503 C08243 C16537 C01018 C08374 C02112 C01725 | | | | | | |

* *P*-value of annotation adjusted for multiple testing using false discovery rate according to Benjamini & Hochberg (1995) doi:10.2307/2346101.

** In set for the present background comparing total Kegg assigned compounds.

**Table C.** Enrichment metabolomic analysis for Enzyme interactions as displayed in MBRole.

| **Enzyme interactions (set: 38 background: 729)** | ***P*-value** | **Adjusted *P*-value*** | **In background** | **In set** | **%**** | **Compounds** |
| --- | --- | --- | --- | --- | --- | --- |
| alanine transaminase | 0.000 | 0.005 | 5 | 4 | 10.5 | C00022 C00025 C00026 C00041 |
| anthranilate synthase | 0.001 | 0.032 | 5 | 3 | 7.9 | C00025 C00064 C00022 |
| asparagine synthase (glutamine-hydrolysing) | 0.001 | 0.032 | 9 | 4 | 10.5 | C00064 C00152 C00025 C00049 |
| aspartate transaminase | 0.001 | 0.032 | 5 | 3 | 7.9 | C00049 C00026 C00025 |
| phenylalanine/tyrosine ammonia-lyase | 0.001 | 0.032 | 5 | 3 | 7.9 | C00811 C00082 C00079 |
| tyrosine transaminase | 0.001 | 0.032 | 5 | 3 | 7.9 | C00082 C00025 C00026 |
| glucose-6-phosphate isomerase | 0.003 | 0.060 | 2 | 2 | 5.3 | C00092 C00085 |
| asparaginyl-tRNA synthase (glutamine-hydrolysing) | 0.004 | 0.070 | 7 | 3 | 7.9 | C00009 C00025 C00064 |
| glutaminyl-tRNA synthase (glutamine-hydrolysing) | 0.004 | 0.070 | 7 | 3 | 7.9 | C00025 C00064 C00009 |
| phosphoribosylformylglycinamidine synthase | 0.006 | 0.097 | 8 | 3 | 7.9 | C00025 C00009 C00064 |
| (R)-3-amino-2-methylpropionate---pyruvate transaminase | 0.015 | 0.098 | 4 | 2 | 5.3 | C00041 C00022 |
| carbamoyl-phosphate synthase (glutamine-hydrolysing) | 0.009 | 0.098 | 9 | 3 | 7.9 | C00064 C00025 C00009 |
| fructokinase | 0.015 | 0.098 | 4 | 2 | 5.3 | C00085 C00095 |
| fructose-2, 6-bisphosphate 2-phosphatase | 0.015 | 0.098 | 4 | 2 | 5.3 | C00085 C00009 |
| fructose-bisphosphatase | 0.015 | 0.098 | 4 | 2 | 5.3 | C00085 C00009 |
| fumarate hydratase | 0.008 | 0.098 | 3 | 2 | 5.3 | C00149 C00122 |
| glutamate decarboxylase | 0.015 | 0.098 | 4 | 2 | 5.3 | C00334 C00025 |
| glutamate N-acetyltransferase | 0.015 | 0.098 | 4 | 2 | 5.3 | C00025 C00077 |
| glutamate---ammonia ligase | 0.012 | 0.098 | 10 | 3 | 7.9 | C00009 C00025 C00064 |
| glycerol kinase | 0.015 | 0.098 | 4 | 2 | 5.3 | C00093 C00116 |
| ornithine carbamoyltransferase | 0.015 | 0.098 | 4 | 2 | 5.3 | C00077 C00009 |
| ornithine decarboxylase | 0.015 | 0.098 | 4 | 2 | 5.3 | C00134 C00077 |
| phosphorylase | 0.009 | 0.098 | 9 | 3 | 7.9 | C00009 C00198 C00092 |
| sucrose synthase | 0.015 | 0.098 | 4 | 2 | 5.3 | C00089 C00095 |
| glutamate synthase (ferredoxin) | 0.016 | 0.103 | 11 | 3 | 7.9 | C00025 C00064 C00026 |
| alanine---glyoxylate transaminase | 0.024 | 0.119 | 5 | 2 | 5.3 | C00022 C00041 |
| branched-chain-amino-acid transaminase | 0.024 | 0.119 | 5 | 2 | 5.3 | C00025 C00026 |
| histidinol-phosphate transaminase | 0.024 | 0.119 | 5 | 2 | 5.3 | C00025 C00026 |
| L-lactate dehydrogenase | 0.024 | 0.119 | 5 | 2 | 5.3 | C00022 C00186 |
| malate dehydrogenase (oxaloacetate-decarboxylating) (NADP+) | 0.024 | 0.119 | 5 | 2 | 5.3 | C00149 C00022 |
| serine---pyruvate transaminase | 0.024 | 0.119 | 5 | 2 | 5.3 | C00041 C00022 |
| threonine synthase | 0.024 | 0.119 | 5 | 2 | 5.3 | C00188 C00009 |
| adenylosuccinate synthase | 0.035 | 0.142 | 6 | 2 | 5.3 | C00049 C00009 |
| amidophosphoribosyltransferase | 0.035 | 0.142 | 6 | 2 | 5.3 | C00064 C00025 |
| phosphoribosylaminoimidazolesuccinocarboxamide synthase | 0.035 | 0.142 | 6 | 2 | 5.3 | C00009 C00049 |
| phosphoserine transaminase | 0.035 | 0.142 | 6 | 2 | 5.3 | C00025 C00026 |
| pyruvate & phosphate dikinase | 0.035 | 0.142 | 6 | 2 | 5.3 | C00022 C00009 |
| succinate---CoA ligase (ADP-forming) | 0.035 | 0.142 | 6 | 2 | 5.3 | C00009 C00042 |
| succinate---CoA ligase (GDP-forming) | 0.035 | 0.142 | 6 | 2 | 5.3 | C00042 C00009 |
| aspartate carbamoyltransferase | 0.047 | 0.178 | 7 | 2 | 5.3 | C00009 C00049 |
| ATP citrate synthase | 0.047 | 0.178 | 7 | 2 | 5.3 | C00158 C00009 |
| glutamate---cysteine ligase | 0.047 | 0.178 | 7 | 2 | 5.3 | C00009 C00025 |
| procollagen-proline dioxygenase | 0.061 | 0.220 | 8 | 2 | 5.3 | C00042 C00026 |
| succinate dehydrogenase (ubiquinone) | 0.061 | 0.220 | 8 | 2 | 5.3 | C00122 C00042 |
| glutamate dehydrogenase [NAD(P)+] | 0.076 | 0.261 | 9 | 2 | 5.3 | C00025 C00026 |
| GMP synthase (glutamine-hydrolysing) | 0.076 | 0.261 | 9 | 2 | 5.3 | C00064 C00025 |
| 1-deoxy-D-xylulose-5-phosphate synthase | 1.000 | 1.000 | 4 | 1 | 2.6 | C00022 |
| 3'(2'),5'-bisphosphate nucleotidase | 1.000 | 1.000 | 4 | 1 | 2.6 | C00009 |
| 3-dehydroquinate synthase | 1.000 | 1.000 | 4 | 1 | 2.6 | C00009 |
| 3-deoxy-7-phosphoheptulonate synthase | 1.000 | 1.000 | 6 | 1 | 2.6 | C00009 |
| 3-deoxy-8-phosphooctulonate synthase | 1.000 | 1.000 | 5 | 1 | 2.6 | C00009 |
| 4-nitrophenylphosphatase | 1.000 | 1.000 | 4 | 1 | 2.6 | C00009 |
| 5'-nucleotidase | 1.000 | 1.000 | 4 | 1 | 2.6 | C00009 |
| 6-phosphofructokinase | 1.000 | 1.000 | 6 | 1 | 2.6 | C00085 |
| acetolactate synthase | 1.000 | 1.000 | 9 | 1 | 2.6 | C00022 |
| acetyl-CoA carboxylase | 1.000 | 1.000 | 8 | 1 | 2.6 | C00009 |
| acetylornithine deacetylase | 1.000 | 1.000 | 4 | 1 | 2.6 | C00077 |
| acireductone synthase | 1.000 | 1.000 | 5 | 1 | 2.6 | C00009 |
| aconitate hydratase | 1.000 | 1.000 | 7 | 1 | 2.6 | C00158 |
| acylphosphatase | 1.000 | 1.000 | 3 | 1 | 2.6 | C00009 |
| adenosine deaminase | 1.000 | 1.000 | 6 | 1 | 2.6 | C00212 |
| adenosylhomocysteinase | 1.000 | 1.000 | 4 | 1 | 2.6 | C00212 |
| adenosylmethionine decarboxylase | 1.000 | 1.000 | 3 | 1 | 2.6 | C00022 |
| adenylosuccinate lyase | 1.000 | 1.000 | 5 | 1 | 2.6 | C00122 |
| alanine---tRNA ligase | 1.000 | 1.000 | 6 | 1 | 2.6 | C00041 |
| alpha,alpha-trehalase | 1.000 | 1.000 | 3 | 1 | 2.6 | C00031 |
| alpha,alpha-trehalose-phosphate synthase (UDP-forming) | 1.000 | 1.000 | 4 | 1 | 2.6 | C00092 |
| amino-acid N-acetyltransferase | 1.000 | 1.000 | 4 | 1 | 2.6 | C00025 |
| arginase | 1.000 | 1.000 | 4 | 1 | 2.6 | C00077 |
| argininosuccinate lyase | 1.000 | 1.000 | 3 | 1 | 2.6 | C00122 |
| argininosuccinate synthase | 1.000 | 1.000 | 6 | 1 | 2.6 | C00049 |
| arsenite-transporting ATPase | 1.000 | 1.000 | 5 | 1 | 2.6 | C00009 |
| asparagine---tRNA ligase | 1.000 | 1.000 | 6 | 1 | 2.6 | C00152 |
| aspartate kinase | 1.000 | 1.000 | 4 | 1 | 2.6 | C00049 |
| aspartate-semialdehyde dehydrogenase | 1.000 | 1.000 | 6 | 1 | 2.6 | C00009 |
| aspartate---tRNA ligase | 1.000 | 1.000 | 6 | 1 | 2.6 | C00049 |
| biotin carboxylase | 1.000 | 1.000 | 6 | 1 | 2.6 | C00009 |
| Ca2+-transporting ATPase | 1.000 | 1.000 | 6 | 1 | 2.6 | C00009 |
| Cd2+-exporting ATPase | 1.000 | 1.000 | 5 | 1 | 2.6 | C00009 |
| CDP-diacylglycerol---glycerol-3-phosphate 3-phosphatidyltransferase | 1.000 | 1.000 | 4 | 1 | 2.6 | C00093 |
| chorismate synthase | 1.000 | 1.000 | 3 | 1 | 2.6 | C00009 |
| citrate (Si)-synthase | 1.000 | 1.000 | 5 | 1 | 2.6 | C00158 |
| CTP synthase | 1.000 | 1.000 | 6 | 1 | 2.6 | C00009 |
| Cu2+-exporting ATPase | 1.000 | 1.000 | 5 | 1 | 2.6 | C00009 |
| cyanoalanine nitrilase | 1.000 | 1.000 | 4 | 1 | 2.6 | C00049 |
| cystathionine beta-lyase | 1.000 | 1.000 | 6 | 1 | 2.6 | C00022 |
| cysteine desulfurase | 1.000 | 1.000 | 4 | 1 | 2.6 | C00041 |
| diaminopimelate decarboxylase | 1.000 | 1.000 | 4 | 1 | 2.6 | C00047 |
| dihydrodipicolinate synthase | 1.000 | 1.000 | 4 | 1 | 2.6 | C00022 |
| diphosphomevalonate decarboxylase | 1.000 | 1.000 | 6 | 1 | 2.6 | C00009 |
| fumarylacetoacetase | 1.000 | 1.000 | 4 | 1 | 2.6 | C00122 |
| gluconokinase | 1.000 | 1.000 | 4 | 1 | 2.6 | C00257 |
| glucose-6-phosphate dehydrogenase | 1.000 | 1.000 | 6 | 1 | 2.6 | C00092 |
| glutamate 5-kinase | 1.000 | 1.000 | 4 | 1 | 2.6 | C00025 |
| glutamate-5-semialdehyde dehydrogenase | 1.000 | 1.000 | 6 | 1 | 2.6 | C00009 |
| glutamate---tRNA ligase | 1.000 | 1.000 | 6 | 1 | 2.6 | C00025 |
| glutamine---tRNA ligase | 1.000 | 1.000 | 6 | 1 | 2.6 | C00064 |
| glutathione synthase | 1.000 | 1.000 | 7 | 1 | 2.6 | C00009 |
| glyceraldehyde-3-phosphate dehydrogenase (phosphorylating) | 1.000 | 1.000 | 6 | 1 | 2.6 | C00009 |
| glycerol-3-phosphate dehydrogenase | 1.000 | 1.000 | 4 | 1 | 2.6 | C00093 |
| glycerol-3-phosphate dehydrogenase (NAD+) | 1.000 | 1.000 | 5 | 1 | 2.6 | C00093 |
| glycerol-3-phosphate O-acyltransferase | 1.000 | 1.000 | 4 | 1 | 2.6 | C00093 |
| glycerophosphodiester phosphodiesterase | 1.000 | 1.000 | 4 | 1 | 2.6 | C00093 |
| H+-transporting two-sector ATPase | 1.000 | 1.000 | 5 | 1 | 2.6 | C00009 |
| indole-3-glycerol-phosphate synthase | 1.000 | 1.000 | 5 | 1 | 2.6 | C00022 |
| inorganic diphosphatase | 1.000 | 1.000 | 4 | 1 | 2.6 | C00009 |
| inositol-3-phosphate synthase | 1.000 | 1.000 | 3 | 1 | 2.6 | C00092 |
| inositol-phosphate phosphatase | 1.000 | 1.000 | 4 | 1 | 2.6 | C00009 |
| isocitrate dehydrogenase (NADP+) | 1.000 | 1.000 | 7 | 1 | 2.6 | C00026 |
| isoleucine---tRNA ligase | 1.000 | 1.000 | 6 | 1 | 2.6 | C00407 |
| L-aspartate oxidase | 1.000 | 1.000 | 5 | 1 | 2.6 | C00049 |
| lysine---tRNA ligase | 1.000 | 1.000 | 6 | 1 | 2.6 | C00047 |
| magnesium chelatase | 1.000 | 1.000 | 8 | 1 | 2.6 | C00009 |
| malate dehydrogenase | 1.000 | 1.000 | 5 | 1 | 2.6 | C00149 |
| malate synthase | 1.000 | 1.000 | 5 | 1 | 2.6 | C00149 |
| mannitol-1-phosphate 5-dehydrogenase | 1.000 | 1.000 | 5 | 1 | 2.6 | C00085 |
| mannose-6-phosphate isomerase | 1.000 | 1.000 | 3 | 1 | 2.6 | C00085 |
| methionine adenosyltransferase | 1.000 | 1.000 | 6 | 1 | 2.6 | C00009 |
| methylcrotonoyl-CoA carboxylase | 1.000 | 1.000 | 7 | 1 | 2.6 | C00009 |
| microtubule-severing ATPase | 1.000 | 1.000 | 4 | 1 | 2.6 | C00009 |
| N4-(beta-N-acetylglucosaminyl)-L-asparaginase | 1.000 | 1.000 | 4 | 1 | 2.6 | C00049 |
| Na+/K+-exchanging ATPase | 1.000 | 1.000 | 9 | 1 | 2.6 | C00009 |
| N-acetyl-gamma-glutamyl-phosphate reductase | 1.000 | 1.000 | 6 | 1 | 2.6 | C00009 |
| oleoyl-[acyl-carrier-protein] hydrolase | 1.000 | 1.000 | 4 | 1 | 2.6 | C00712 |
| ornithine aminotransferase | 1.000 | 1.000 | 5 | 1 | 2.6 | C00077 |
| oxoglutarate dehydrogenase (succinyl-transferring) | 1.000 | 1.000 | 4 | 1 | 2.6 | C00026 |
| pantoate---beta-alanine ligase | 1.000 | 1.000 | 6 | 1 | 2.6 | C00099 |
| phenylalanine---tRNA ligase | 1.000 | 1.000 | 6 | 1 | 2.6 | C00079 |
| phosphatidylserine decarboxylase | 1.000 | 1.000 | 5 | 1 | 2.6 | C00022 |
| phosphoenolpyruvate carboxylase | 1.000 | 1.000 | 5 | 1 | 2.6 | C00009 |
| phosphoglycerate dehydrogenase | 1.000 | 1.000 | 7 | 1 | 2.6 | C00026 |
| phospholipid-translocating ATPase | 1.000 | 1.000 | 5 | 1 | 2.6 | C00009 |
| phosphoprotein phosphatase | 1.000 | 1.000 | 12 | 1 | 2.6 | C00009 |
| phosphoribosylamine---glycine ligase | 1.000 | 1.000 | 6 | 1 | 2.6 | C00009 |
| phosphoserine phosphatase | 1.000 | 1.000 | 5 | 1 | 2.6 | C00009 |
| polynucleotide 3'-phosphatase | 1.000 | 1.000 | 4 | 1 | 2.6 | C00009 |
| polynucleotide 5'-phosphatase | 1.000 | 1.000 | 4 | 1 | 2.6 | C00009 |
| polyribonucleotide nucleotidyltransferase | 1.000 | 1.000 | 3 | 1 | 2.6 | C00009 |
| protein-synthesizing GTPase | 1.000 | 1.000 | 4 | 1 | 2.6 | C00009 |
| protein-tyrosine-phosphatase | 1.000 | 1.000 | 4 | 1 | 2.6 | C00009 |
| pyruvate dehydrogenase (acetyl-transferring) | 1.000 | 1.000 | 4 | 1 | 2.6 | C00022 |
| pyruvate kinase | 1.000 | 1.000 | 5 | 1 | 2.6 | C00022 |
| ribokinase | 1.000 | 1.000 | 4 | 1 | 2.6 | C00121 |
| selenocysteine lyase | 1.000 | 1.000 | 6 | 1 | 2.6 | C00041 |
| shikimate dehydrogenase | 1.000 | 1.000 | 5 | 1 | 2.6 | C00493 |
| shikimate kinase | 1.000 | 1.000 | 3 | 1 | 2.6 | C00493 |
| spermidine synthase | 1.000 | 1.000 | 3 | 1 | 2.6 | C00134 |
| succinate-semialdehyde dehydrogenase [NAD(P)+] | 1.000 | 1.000 | 8 | 1 | 2.6 | C00042 |
| sucrose-phosphate synthase | 1.000 | 1.000 | 4 | 1 | 2.6 | C00085 |
| threonine aldolase | 1.000 | 1.000 | 4 | 1 | 2.6 | C00188 |
| threonine ammonia-lyase | 1.000 | 1.000 | 4 | 1 | 2.6 | C00188 |
| transaldolase | 1.000 | 1.000 | 4 | 1 | 2.6 | C00085 |
| trans-cinnamate 4-monooxygenase | 1.000 | 1.000 | 8 | 1 | 2.6 | C00811 |
| trehalose-phosphatase | 1.000 | 1.000 | 4 | 1 | 2.6 | C00009 |
| tryptophan synthase | 1.000 | 1.000 | 7 | 1 | 2.6 | C00078 |
| tryptophan---tRNA ligase | 1.000 | 1.000 | 5 | 1 | 2.6 | C00078 |
| tyrosine---tRNA ligase | 1.000 | 1.000 | 6 | 1 | 2.6 | C00082 |
| xylose isomerase | 1.000 | 1.000 | 2 | 1 | 2.6 | C00181 |
| Zn2+-exporting ATPase | 1.000 | 1.000 | 5 | 1 | 2.6 | C00009 |
| Compounds with no annotations (45): C05659 C08240 C01389 C01601 C00408 C00180 C08243 C06427 C00259 C00185 C00189 C08362 C01725 C05437 C00503 C01571 C06425 C01451 C01530 C01018 C01384 C06424 C00474 C00156 C00192 C00208 C05442 C00296 C01694 C16537 C01904 C01595 C08374 C00249 C01879 C01235 C00379 C05422 C02989 C01753 C05401 C00387 C02679 C02112 C02457 | | | | |  |  |

* *P*-value of annotation adjusted for multiple testing using false discovery rate according to Benjamini & Hochberg (1995) doi:10.2307/2346101.

** In set for the present background comparing total Kegg assigned compounds.

**Table D.** Enrichment metabolomic analysis for Biological role as displayed in MBRole.

| **Biological role (set: 44 background: 1117)** | ***P*-value** | **Adjusted *P*-value*** | **In background** | **In set** | **%**** | **Compounds** |
| --- | --- | --- | --- | --- | --- | --- |
| Common amino acids | 0.000 | 0.000 | 20 | 11 | 25 | C00041 C00078 C00049 C00152 C00064 C00082 C00188 C00407 C00047 C00025 C00079 |
| Peptides | 0.000 | 0.000 | 54 | 16 | 36.4 | C00407 C00152 C00077 C00025 C00082 C00064 C00041 C00079 C00099 C00188 C00334 C00189 C00134 C00049 C00047 C00078 |
| Amino acids | 0.000 | 0.000 | 45 | 14 | 31.8 | C00152 C00025 C00049 C00077 C00099 C00078 C00407 C00334 C00188 C00079 C00041 C00047 C00082 C00064 |
| Fatty acids | 0.000 | 0.000 | 25 | 9 | 20.5 | C06425 C00249 C01595 C06427 C02679 C06424 C00712 C01571 C01530 |
| FA0101 Straight chain fatty acids | 0.000 | 0.000 | 13 | 6 | 13.6 | C01571 C06424 C06425 C00249 C02679 C01530 |
| Saturated fatty acids | 0.000 | 0.000 | 13 | 6 | 13.6 | C01530 C06425 C01571 C06424 C02679 C00249 |
| FA01 Fatty Acids and Conjugates | 0.000 | 0.000 | 62 | 11 | 25 | C00249 C00712 C01530 C08362 C06427 C01571 C06424 C02679 C01595 C00334 C06425 |
| Carbohydrates | 0.000 | 0.000 | 43 | 9 | 20.5 | C00089 C00208 C00185 C00095 C00031 C00121 C00257 C00259 C00181 |
| Lipids | 0.000 | 0.000 | 67 | 11 | 25 | C01571 C01595 C00712 C00116 C02679 C00249 C06427 C06425 C01694 C06424 C01530 |
| Aldoses | 0.000 | 0.001 | 9 | 4 | 9.1 | C00181 C00259 C00031 C00121 |
| Amines | 0.001 | 0.002 | 11 | 4 | 9.1 | C00134 C00189 C00099 C00334 |
| Biogenic amines | 0.001 | 0.002 | 11 | 4 | 9.1 | C00134 C00334 C00189 C00099 |
| Disaccharides | 0.001 | 0.004 | 6 | 3 | 6.8 | C00185 C00089 C00208 |
| Oligosaccharides | 0.001 | 0.004 | 6 | 3 | 6.8 | C00185 C00089 C00208 |
| Monosaccharides | 0.002 | 0.008 | 37 | 6 | 13.6 | C00181 C00257 C00121 C00259 C00095 C00031 |
| FA0103 Unsaturated fatty acids | 0.003 | 0.010 | 17 | 4 | 9.1 | C01595 C06427 C08362 C00712 |
| ST0105 Fungal sterols and derivatives | 0.004 | 0.013 | 3 | 2 | 4.5 | C01694 C05437 |
| Ribonucleosides | 0.009 | 0.023 | 4 | 2 | 4.5 | C00387 C00212 |
| Neurotransmitters | 0.010 | 0.023 | 12 | 3 | 6.8 | C00334 C00049 C00025 |
| Unsaturated fatty acids | 0.010 | 0.023 | 12 | 3 | 6.8 | C06427 C01595 C00712 |
| Monounsaturated fatty acids | 0.014 | 0.032 | 5 | 2 | 4.5 | C00712 C08362 |
| ST01 Sterols | 0.014 | 0.032 | 25 | 4 | 9.1 | C05442 C01753 C05437 C01694 |
| ST0103 Phytosterols and derivatives | 0.037 | 0.076 | 8 | 2 | 4.5 | C01753 C05442 |
| Nucleosides | 0.046 | 0.092 | 9 | 2 | 4.5 | C00387 C00212 |
| Other amino acids | 0.072 | 0.138 | 25 | 3 | 6.8 | C00334 C00099 C00077 |
| Polyunsaturated fatty acids | 0.078 | 0.144 | 12 | 2 | 4.5 | C01595 C06427 |
| FA Fatty acyls | 0.128 | 0.228 | 195 | 11 | 25 | C00249 C06424 C02679 C08362 C01530 C00334 C01571 C06427 C00712 C01595 C06425 |
| Hormones and transmitters | 0.156 | 0.258 | 35 | 3 | 6.8 | C00334 C00049 C00025 |
| Sterols and steroids | 0.156 | 0.258 | 18 | 2 | 4.5 | C01753 C05442 |
| Triterpenoids (C30) and related compounds | 0.214 | 0.342 | 22 | 2 | 4.5 | C01753 C05442 |
| ST Sterol Lipids | 0.233 | 0.360 | 63 | 4 | 9.1 | C01753 C01694 C05442 C05437 |
| Nucleic acids | 0.562 | 0.844 | 47 | 2 | 4.5 | C00212 C00387 |
| Alkaloids | 1.000 | 1.000 | 200 | 1 | 2.3 | C00408 |
| Alkaloids derived from lysine | 1.000 | 1.000 | 18 | 1 | 2.3 | C00408 |
| Biogenic amines | 1.000 | 1.000 | 8 | 1 | 2.3 | C00334 |
| FA0110 Amino fatty acids | 1.000 | 1.000 | 8 | 1 | 2.3 | C00334 |
| Fats | 1.000 | 1.000 | 4 | 1 | 2.3 | C00116 |
| Glycerols | 1.000 | 1.000 | 1 | 1 | 2.3 | C00116 |
| Ketoses | 1.000 | 1.000 | 9 | 1 | 2.3 | C00095 |
| Monolignols | 1.000 | 1.000 | 37 | 1 | 2.3 | C00811 |
| Phenylpropanoids and related compounds | 1.000 | 1.000 | 140 | 1 | 2.3 | C00811 |
| Piperidine alkaloids | 1.000 | 1.000 | 15 | 1 | 2.3 | C00408 |
| ST03 Secosteroids | 1.000 | 1.000 | 6 | 1 | 2.3 | C01694 |
| ST0301 Vitamin D2 and derivatives | 1.000 | 1.000 | 3 | 1 | 2.3 | C01694 |
| Steroids | 1.000 | 1.000 | 4 | 1 | 2.3 | C01694 |
| Sterols | 1.000 | 1.000 | 2 | 1 | 2.3 | C01694 |
| Terpenoids | 0.993 | 1.000 | 165 | 2 | 4.5 | C05442 C01753 |
| Uronic acids | 1.000 | 1.000 | 8 | 1 | 2.3 | C00257 |
| Compounds with no annotations (39): C05659 C08240 C01389 C01601 C00026 C00180 C08243 C00186 C01725 C00503 C00085 C00149 C01451 C01384 C01018 C00042 C00022 C00493 C00474 C00156 C00192 C00296 C01904 C16537 C08374 C00198 C01879 C01235 C00379 C02989 C05422 C05401 C00158 C00092 C00093 C00122 C02112 C00009 C02457 | | | | | |  |

* *P*-value of annotation adjusted for multiple testing using false discovery rate according to Benjamini & Hochberg (1995) doi:10.2307/2346101.

** In set for the present background comparing total Kegg assigned compounds.

**Table E.** Enrichment metabolomic analysis for Chemical groups as displayed in MBRole.

| **Chemical groups (set: 64 background: 2902)** | ***P*-value** | **Adjusted *P*-value*** | **In background** | **In set** | **%**** | **Compounds** |
| --- | --- | --- | --- | --- | --- | --- |
| carboxylicacid | 0.000 | 0.000 | 1084 | 41 | 64.1 | C00712 C06425 C00077 C00079 C00064 C00078 C00186 C02989 C06424 C00022 C00249 C00408 C00047 C00180 C01879 C00158 C00152 C00188 C00122 C00049 C00041 C00156 C08362 C02679 C06427 C00493 C00026 C01595 C00334 C01384 C00407 C01571 C00811 C01530 C00149 C00042 C00082 C00099 C00257 C00025 C00296 |
| alpha-aminoacid | 0.000 | 0.001 | 200 | 14 | 21.9 | C00041 C00025 C00078 C00049 C00064 C00077 C00082 C00407 C00047 C00188 C00408 C02989 C00152 C00079 |
| prim.aliphat.amine | 0.000 | 0.004 | 354 | 18 | 28.1 | C00047 C00152 C00064 C00188 C00077 C00099 C00025 C00189 C05659 C02989 C00049 C00082 C00079 C00407 C00134 C00041 C00334 C00078 |
| prim.alcohol | 0.025 | 0.164 | 439 | 16 | 25 | C00116 C05422 C00198 C01451 C00089 C05401 C01904 C00474 C01235 C02457 C00093 C00257 C00387 C00212 C00379 C00189 |
| alpha-hydroxyacid | 0.067 | 0.351 | 100 | 5 | 7.8 | C00296 C00158 C00149 C00186 C00257 |
| 1;2-aminoalcohol | 0.559 | 1.000 | 84 | 2 | 3.1 | C00189 C00188 |
| 1;2-diol | 0.306 | 1.000 | 631 | 16 | 25 | C01235 C00379 C00093 C00387 C00212 C00116 C00474 C01451 C05401 C00296 C00089 C00257 C00198 C05422 C01904 C00493 |
| acetal | 0.747 | 1.000 | 226 | 4 | 6.2 | C05401 C00089 C01235 C01451 |
| alkene | 0.998 | 1.000 | 989 | 12 | 18.8 | C08362 C01694 C00712 C00493 C00811 C01753 C00122 C05437 C01595 C05442 C01384 C06427 |
| alkylarylether | 1.000 | 1.000 | 202 | 1 | 1.6 | C05659 |
| aromaticcompound | 1.000 | 1.000 | 1406 | 10 | 15.6 | C00387 C00079 C00082 C00156 C05659 C00212 C01451 C00180 C00078 C00811 |
| carboxylicacidester | 0.964 | 1.000 | 224 | 2 | 3.1 | C00198 C05422 |
| heterocycliccompound | 1.000 | 1.000 | 1417 | 12 | 18.8 | C00387 C05659 C01235 C00212 C01451 C00078 C00089 C05422 C05401 C00198 C01879 C00408 |
| hydroxylamine | 1.000 | 1.000 | 11 | 1 | 1.6 | C00192 |
| iminohetarene | 1.000 | 1.000 | 111 | 1 | 1.6 | C00387 |
| ketone | 1.000 | 1.000 | 605 | 3 | 4.7 | C00026 C00022 C05422 |
| lactone | 0.559 | 1.000 | 84 | 2 | 3.1 | C00198 C05422 |
| phenol | 1.000 | 1.000 | 763 | 4 | 6.2 | C00082 C00156 C00387 C00811 |
| phosphoricacid | 1.000 | 1.000 | 1 | 1 | 1.6 | C00009 |
| phosphoricacidderiv. | 1.000 | 1.000 | 625 | 2 | 3.1 | C00093 C00009 |
| phosphoricacidester | 1.000 | 1.000 | 607 | 1 | 1.6 | C00093 |
| prim.aromat.amine | 1.000 | 1.000 | 254 | 1 | 1.6 | C00212 |
| sec.alcohol | 0.927 | 1.000 | 1278 | 23 | 35.9 | C01694 C01235 C00493 C05401 C01451 C00379 C00149 C01904 C01753 C00474 C00198 C00257 C05442 C00212 C00296 C00093 C00089 C00188 C00387 C05437 C00116 C00186 C05422 |
| sec.aliphat.amine | 1.000 | 1.000 | 77 | 1 | 1.6 | C00408 |
| sulfoxide | 1.000 | 1.000 | 1 | 1 | 1.6 | C02989 |
| tert.alcohol | 0.940 | 1.000 | 198 | 2 | 3.1 | C00158 C00296 |
| Compounds with no annotations (19): C08240 C01389 C01601 C00181 C08243 C00208 C00259 C00185 C16537 C08374 C01725 C00503 C00085 C00095 C00031 C00092 C01018 C00121 C02112 | | | | | |  |

* *P*-value of annotation adjusted for multiple testing using false discovery rate according to Benjamini & Hochberg (1995) doi:10.2307/2346101.

** In set for the present background comparing total Kegg assigned compounds.

**Table F.** Enrichment metabolomic analysis for Other interactions as displayed in MBRole.

| **Other interactions (set: 28 background: 224)** | ***P*-value** | **Adjusted *P*-value*** | **In background** | **In set** | **%**** | **Compounds** |
| --- | --- | --- | --- | --- | --- | --- |
| ABC Transporters, Prokaryotic Type | 0.000 | 0.000 | 69 | 21 | 75 | C00031 C00134 C00049 C00185 C00208 C00009 C00093 C00079 C00181 C00188 C00064 C00089 C00121 C00025 C00047 C00041 C00259 C00077 C05401 C00407 C00095 |
| Phosphate and amino acid transporters | 0.000 | 0.001 | 21 | 10 | 35.7 | C00079 C00047 C00077 C00064 C00407 C00009 C00049 C00188 C00025 C00041 |
| Simple sugar transporters | 0.000 | 0.004 | 9 | 6 | 21.4 | C00031 C00095 C05401 C00181 C00121 C00259 |
| Metabotropic glutamate family | 0.001 | 0.016 | 15 | 7 | 25 | C00077 C00041 C00334 C00047 C00025 C00078 C00064 |
| D-Xylose transporter | 0.015 | 0.111 | 2 | 2 | 7.1 | C00121 C00181 |
| Glucose/arabinose transporter | 0.015 | 0.111 | 5 | 3 | 10.7 | C00181 C00031 C00095 |
| Glutamate (ionotropic), non-NMDA | 0.015 | 0.111 | 2 | 2 | 7.1 | C00025 C00049 |
| Glutamate/aspartate transporter | 0.015 | 0.111 | 2 | 2 | 7.1 | C00025 C00049 |
| GPRC6A, G protein-coupled receptor, family C, group 6, member A | 0.010 | 0.111 | 8 | 4 | 14.3 | C00047 C00077 C00041 C00064 |
| GRIA, glutamate receptor, ionotropic, AMPA | 0.015 | 0.111 | 2 | 2 | 7.1 | C00025 C00049 |
| GRIK, glutamate receptor, ionotropic, kainate | 0.015 | 0.111 | 2 | 2 | 7.1 | C00025 C00049 |
| Orphan GPCR6 | 0.010 | 0.111 | 8 | 4 | 14.3 | C00047 C00064 C00077 C00041 |
| Basic organic compound transporters | 0.023 | 0.154 | 37 | 9 | 32.1 | C00185 C00089 C00093 C00208 C00095 C00134 C00031 C00259 C00181 |
| alpha-Glucoside transporter | 0.042 | 0.202 | 3 | 2 | 7.1 | C00208 C00089 |
| Fructose transporter | 0.042 | 0.202 | 3 | 2 | 7.1 | C00121 C00095 |
| Lysine/arginine/ornithine transporter | 0.042 | 0.202 | 3 | 2 | 7.1 | C00047 C00077 |
| Methyl-galactoside transporter | 0.042 | 0.202 | 3 | 2 | 7.1 | C00031 C05401 |
| Multiple sugar transporter | 0.044 | 0.202 | 7 | 3 | 10.7 | C00181 C00031 C00259 |
| Trehalose/maltose transporter | 0.042 | 0.202 | 3 | 2 | 7.1 | C00089 C00208 |
| Neutral amino acid transporter | 0.064 | 0.281 | 8 | 3 | 10.7 | C00041 C00064 C00079 |
| Branched-chain amino acid transporter | 0.119 | 0.436 | 5 | 2 | 7.1 | C00188 C00407 |
| Glutamate (ionotropic), NMDA | 0.119 | 0.436 | 5 | 2 | 7.1 | C00025 C00049 |
| Glutamate-gated cation channels | 0.119 | 0.436 | 5 | 2 | 7.1 | C00025 C00049 |
| GRIN, glutamate receptor, ionotropic, N-methyl D-aspartate | 0.119 | 0.436 | 5 | 2 | 7.1 | C00049 C00025 |
| GLRA1/4, glycine receptor, alpha 1/4 | 0.165 | 0.517 | 6 | 2 | 7.1 | C00334 C00099 |
| GLRA2, glycine receptor, alpha 2 | 0.165 | 0.517 | 6 | 2 | 7.1 | C00334 C00099 |
| GLRA3, glycine receptor, alpha 3 | 0.165 | 0.517 | 6 | 2 | 7.1 | C00099 C00334 |
| Glycine | 0.165 | 0.517 | 6 | 2 | 7.1 | C00334 C00099 |
| GABA-A | 0.321 | 0.943 | 16 | 3 | 10.7 | C00099 C00334 C00212 |
| GABRA, gamma-aminobutyric acid (GABA) A receptor, alpha | 0.321 | 0.943 | 16 | 3 | 10.7 | C00099 C00212 C00334 |
| ABC-2 type and other transporters | 1.000 | 1.000 | 7 | 1 | 3.6 | C00134 |
| Adenosine | 1.000 | 1.000 | 3 | 1 | 3.6 | C00212 |
| ADORA1, adenosine A1 receptor | 1.000 | 1.000 | 3 | 1 | 3.6 | C00212 |
| ADORA2A, adenosine A2a receptor | 1.000 | 1.000 | 3 | 1 | 3.6 | C00212 |
| ADORA2B, adenosine A2b receptor | 1.000 | 1.000 | 1 | 1 | 3.6 | C00212 |
| ADORA3, adenosine A3 receptor | 1.000 | 1.000 | 1 | 1 | 3.6 | C00212 |
| Antibiotic transporter | 1.000 | 1.000 | 4 | 1 | 3.6 | C00134 |
| Arginine/ornithine transporter | 1.000 | 1.000 | 2 | 1 | 3.6 | C00077 |
| CASR, calcium-sensing receptor | 1.000 | 1.000 | 3 | 1 | 3.6 | C00078 |
| Cellobiose transporter | 1.000 | 1.000 | 2 | 1 | 3.6 | C00185 |
| CYP2 family | 1.000 | 1.000 | 23 | 1 | 3.6 | C02679 |
| CYP2E subfamily | 1.000 | 1.000 | 12 | 1 | 3.6 | C02679 |
| CYP2E1 | 1.000 | 1.000 | 12 | 1 | 3.6 | C02679 |
| CYP4 family | 1.000 | 1.000 | 16 | 1 | 3.6 | C02679 |
| CYP4A subfamily | 1.000 | 1.000 | 4 | 1 | 3.6 | C02679 |
| CYP4A1 | 1.000 | 1.000 | 3 | 1 | 3.6 | C02679 |
| CYP4A11 | 1.000 | 1.000 | 3 | 1 | 3.6 | C02679 |
| CYP4A2 | 1.000 | 1.000 | 1 | 1 | 3.6 | C02679 |
| Cys-loop superfamily | 0.716 | 1.000 | 28 | 3 | 10.7 | C00099 C00334 C00212 |
| D-Allose transporter | 1.000 | 1.000 | 1 | 1 | 3.6 | C00121 |
| Extracellular calcium-sensing | 1.000 | 1.000 | 3 | 1 | 3.6 | C00078 |
| GABA-B | 1.000 | 1.000 | 1 | 1 | 3.6 | C00334 |
| GABBR1, gamma-aminobutyric acid (GABA) B receptor | 1.000 | 1.000 | 1 | 1 | 1 | C00334 |
| Glutamate (metabotropic) | 1.000 | 1.000 | 3 | 1 | 3.6 | C00025 |
| Glutamate transporter | 1.000 | 1.000 | 1 | 1 | 3.6 | C00025 |
| Glutamine transporter | 1.000 | 1.000 | 1 | 1 | 3.6 | C00064 |
| GRM1, glutamate receptor, metabotropic 1 | 1.000 | 1.000 | 2 | 1 | 3.6 | C00025 |
| GRM2, glutamate receptor, metabotropic 2 | 1.000 | 1.000 | 1 | 1 | 3.6 | C00025 |
| GRM3, glutamate receptor, metabotropic 3 | 1.000 | 1.000 | 1 | 1 | 3.6 | C00025 |
| GRM4, glutamate receptor, metabotropic 4 | 1.000 | 1.000 | 2 | 1 | 3.6 | C00025 |
| GRM5, glutamate receptor, metabotropic 5 | 1.000 | 1.000 | 2 | 1 | 3.6 | C00025 |
| GRM6, glutamate receptor, metabotropic 6 | 1.000 | 1.000 | 2 | 1 | 3.6 | C00025 |
| GRM7, glutamate receptor, metabotropic 7 | 1.000 | 1.000 | 2 | 1 | 3.6 | C00025 |
| GRM8, glutamate receptor, metabotropic 8 | 1.000 | 1.000 | 2 | 1 | 3.6 | C00025 |
| Inward rectifier channel (Kir) | 1.000 | 1.000 | 8 | 1 | 3.6 | C00134 |
| K+ channel, KCNB, Kv2.x (Shab) | 1.000 | 1.000 | 1 | 1 | 3.6 | C01595 |
| K+ channel, KCNK, K2px.x | 1.000 | 1.000 | 7 | 1 | 3.6 | C01595 |
| KCNB1, potassium voltage-gated channel, Shab-related subfamily B, member 1 | 1.000 | 1.000 | 1 | 1 | 3.6 | C01595 |
| KCNJ2, potassium inwardly-rectifying channel, subfamily J, member 2 | 1.000 | 1.000 | 4 | 1 | 3.6 | C00134 |
| KCNK10, potassium channel, subfamily K, member 10 | 1.000 | 1.000 | 6 | 1 | 3.6 | C01595 |
| Lactose/L-arabinose transporter | 1.000 | 1.000 | 2 | 1 | 3.6 | C00259 |
| L-Arabinose transporter | 1.000 | 1.000 | 1 | 1 | 3.6 | C00259 |
| Maltose/maltodextrin transporter | 1.000 | 1.000 | 2 | 1 | 3.6 | C00208 |
| Mas proto-oncogene & Mas-related (MRGs) | 1.000 | 1.000 | 1 | 1 | 3.6 | C00099 |
| MRGPRD, MAS-related GPR, member D | 1.000 | 1.000 | 1 | 1 | 3.6 | C00099 |
| Phosphate transporter | 1.000 | 1.000 | 1 | 1 | 3.6 | C00009 |
| Purine / pyrimidine | 1.000 | 1.000 | 15 | 1 | 3.6 | C00042 |
| Putrescine transporter | 1.000 | 1.000 | 1 | 1 | 3.6 | C00134 |
| Related to inward rectifier K+channels | 1.000 | 1.000 | 8 | 1 | 3.6 | C00134 |
| Related to voltage-gated cation channels | 1.000 | 1.000 | 23 | 1 | 3.6 | C00212 |
| Rhodopsin family: other receptors | 0.853 | 1.000 | 35 | 3 | 10.7 | C00042 C00099 C00212 |
| Ribose transporter | 1.000 | 1.000 | 1 | 1 | 3.6 | C00121 |
| Ryanodine receptor (RYR) | 1.000 | 1.000 | 10 | 1 | 3.6 | C00212 |
| RYR, ryanodine receptor | 1.000 | 1.000 | 10 | 1 | 3.6 | C00212 |
| sn-Glycerol 3-phosphate transporter | 1.000 | 1.000 | 1 | 1 | 3.6 | C00093 |
| Spermidine/putrescine transporter | 1.000 | 1.000 | 2 | 1 | 3.6 | C00134 |
| SUCNR1, GPR91, succinate receptor 1 | 1.000 | 1.000 | 1 | 1 | 3.6 | C00042 |
| Voltage-gated cation channels | 1.000 | 1.000 | 14 | 1 | 3.6 | C01595 |
| Compounds with no annotations (55): C00408 C00116 C00180 C00257 C00189 C00186 C01725 C08362 C00503 C00085 C00082 C01451 C00149 C00493 C00152 C00156 C00192 C01694 C00296 C01904 C16537 C08374 C00249 C00198 C01753 C05422 C02989 C00811 C00387 C00158 C00092 C08240 C05659 C01389 C01601 C00026 C08243 C06427 C00712 C05437 C01571 C06425 C01530 C06424 C01384 C01018 C00022 C00474 C05442 C01879 C01235 C00379 C02112 C00122 C02457 | | | | | |  |

* *P*-value of annotation adjusted for multiple testing using false discovery rate according to Benjamini & Hochberg (1995) doi:10.2307/2346101.

** In set for the present background comparing total Kegg assigned compounds.

**Table G.** Group means ± S.E. across standardised data for identified GC peaks grouped per biological group.

| **Class of compounds** | **Subclass** | **n*** | ***P*** | **NAT** | | | **AMF** | | | **AMF+PGPR** | | |
| --- | --- | --- | --- | --- | --- | --- | --- | --- | --- | --- | --- | --- |
| Carbohydrates | Annotated | 38 | 0.44 | 0.04 | ± | 0.18 | -0.15 | ± | 0.12 | 0.11 | ± | 0.15 |
|  | Unannotated | 10 | 0.22 | 0.18 | ± | 0.27 | -0.35 | ± | 0.15 | 0.17 | ± | 0.17 |
| Lipids | Total | 27 | 0.10 | 0.41 | ± | 0.17 | -0.37 | ± | 0.30 | -0.04 | ± | 0.20 |
|  | Sterols | 4 | 0.45 | 0.38 | ± | 0.21 | -0.19 | ± | 0.44 | -0.19 | ± | 0.26 |
|  | Total FA&E | 20 | 0.08 | 0.44 | ± | 0.23 | -0.42 | ± | 0.29 | -0.02 | ± | 0.23 |
|  | Unsaturated FA&E | 7 | 0.31 | 0.31 | ± | 0.29 | -0.45 | ± | 0.27 | 0.14 | ± | 0.37 |
|  | Saturated FA&E | 13 | 0.05 | 0.50 | ± | 0.28 | -0.40 | ± | 0.31 | -0.10 | ± | 0.16 |
| Peptides | Total | 27 | 0.04 | 0.33 | ± | 0.19 | -0.44 | ± | 0.19 | 0.11 | ± | 0.28 |
|  | Amino acids | 21 | 0.04 | 0.41 | ± | 0.23 | -0.54 | ± | 0.18 | 0.13 | ± | 0.35 |
|  | Amines | 6 | 0.62 | 0.03 | ± | 0.12 | -0.10 | ± | 0.25 | 0.08 | ± | 0.11 |
| Others | Organic acids | 24 | 0.85 | 0.01 | ± | 0.10 | -0.08 | ± | 0.26 | 0.06 | ± | 0.19 |
|  | P-containing Compounds | 5 | 1.00 | -0.02 | ± | 0.41 | 0.01 | ± | 0.35 | 0.01 | ± | 0.35 |
|  | Other Compounds | 3 | 0.46 | 0.36 | ± | 0.18 | -0.21 | ± | 0.43 | -0.14 | ± | 0.37 |
| All Compounds |  | 127 | 0.01 | 0.19 | ± | 0.05 | -0.25 | ± | 0.15 | 0.06 | ± | 0.15 |

* n indicates the number of compounds contributing to the relative mean, and *P* is the *P*-value of the ANOVA for that group. Carbohydrates were analysed separately according to KEGG annotation. All compounds include both annotated and unannotated compounds. GC was run with methanol:chloroform:water (5:2:2) extracts from roots of durum wheat grown in the field with natural arbuscular mycorrhizal inoculum (NAT), inoculation with AM fungi (AMF), or inoculation with both AMF and plant growth–promoting rhizobacteria (PGPR). FA&E, fatty acids and their esters.

**Table H.** HILIC-Q-TOF MS identified compounds. Wheat with natural arbuscular mycorrhizal inoculum (NAT), inoculated with AM fungi spores (AMF), or inoculated with both AMF and plant growth–promoting rhizobacteria (AMF+PGPR).

| **Row m/z** | **Row retention time** | **Assigned name** | **Assigned molecular formula** |  | **NAT** | |  | **AMF** | |  | **AMF+PGPR** | |
| --- | --- | --- | --- | --- | --- | --- | --- | --- | --- | --- | --- | --- |
|  |  |  |  |  | **mean** | **S.E.** |  | **mean** | **S.E.** |  | **mean** | **S.E.** |
| 162.111331832826 | 8.14 | Carnitine C0:0 | C7H15NO3 |  | 1297050 | 140113 |  | 1100170 | 139212 |  | 1140694 | 67595 |
| 204.125360147688 | 7.53 | Carnitine C2:0 | C9H17NO4 |  | 2178796 | 104815 |  | 1885182 | 230832 |  | 2007212 | 156289 |
| 218.138724154545 | 7.10 | Carnitine C3:0 | C10H19NO4 |  | 408000 | 54723 |  | 317415 | 84814 |  | 380358 | 79845 |
| 232.152469696014 | 6.68 | Carnitine C4:0 | C11H21NO4 |  | 622782 | 72449 |  | 497447 | 124801 |  | 527594 | 105178 |
| 246.166521744862 | 6.36 | Carnitine C5:0 | C12H23NO4 |  | 563282 | 73084 |  | 346432 | 57413 |  | 379545 | 52015 |
| 258.111401912647 | 8.82 | Glycerophosphocholine | C8H20NO6P |  | 203782 | 44361 |  | 171368 | 41524 |  | 195655 | 32477 |
| 496.342041358028 | 5.19 | LPC 16:0 | C24H50NO7P |  | 83658 | 8661 |  | 59478 | 8663 |  | 65430 | 8940 |
| 520.342671800083 | 4.99 | LPC 18:2 | C26H50NO7P |  | 60394 | 11194 |  | 73237 | 14355 |  | 58032 | 7886 |
| 518.324927309940 | 5.22 | LPC 18:3 | C26H48NO7P |  | 31185 | 7346 |  | 22340 | 2410 |  | 22056 | 1796 |
| 542.324614903292 | 4.98 | LPC 20:5 | C28H48NO7P |  | 48229 | 6946 |  | 50413 | 9412 |  | 37268 | 4153 |
| 454.291432778278 | 3.82 | LPE 16:0 | C21H44NO7P |  | 89413 | 5742 |  | 71313 | 10735 |  | 77703 | 9202 |
| 478.292971167064 | 3.68 | LPE 18:2 | C23H44NO7P |  | 80665 | 6390 |  | 69313 | 7858 |  | 69636 | 6451 |
| 476.279116547599 | 3.85 | LPE 18:3 | C23H42NO7P |  | 30825 | 2498 |  | 27455 | 2162 |  | 26688 | 932 |
| 732.550394519425 | 3.80 | PC 32:1 | C40H78NO8P |  | 83396 | 15219 |  | 68438 | 11726 |  | 93101 | 7376 |
| 730.539874881159 | 3.83 | PC 32:2 | C40H76NO8P |  | 74009 | 9768 |  | 66575 | 9727 |  | 80698 | 5943 |
| 728.531098806654 | 3.84 | PC 32:3 | C40H74NO8P |  | 17088 | 2396 |  | 15823 | 1755 |  | 15766 | 1200 |
| 744.559122955340 | 3.78 | PC 33:2 | C41H78NO8P |  | 345765 | 55456 |  | 335022 | 29798 |  | 361659 | 40240 |
| 742.547053951094 | 3.76 | PC 33:3 | C41H76NO8P |  | 135692 | 30928 |  | 127711 | 12656 |  | 122462 | 10798 |
| 758.568223571777 | 3.75 | PC 34:2 | C42H80NO8P |  | 3728031 | 456719 |  | 3841986 | 343719 |  | 4075886 | 358470 |
| 756.553636600625 | 3.77 | PC 34:3 | C42H78NO8P |  | 1976427 | 240899 |  | 1933897 | 215946 |  | 2015736 | 101951 |
| 754.533900546512 | 3.75 | PC 34:4 | C42H76NO8P |  | 109454 | 25134 |  | 76399 | 13605 |  | 98631 | 11812 |
| 752.521186909934 | 3.76 | PC 34:5 | C42H74NO8P |  | 33321 | 11286 |  | 20275 | 4364 |  | 31797 | 6777 |
| 772.579167569223 | 3.72 | PC 35:2 | C43H82NO8P |  | 94015 | 15304 |  | 79975 | 6294 |  | 84955 | 5128 |
| 770.569566527624 | 3.72 | PC 35:3 | C43H80NO8P |  | 94741 | 6008 |  | 86198 | 9214 |  | 83416 | 2992 |
| 768.558704555256 | 3.71 | PC 35:4 | C43H78NO8P |  | 61051 | 5841 |  | 61806 | 5351 |  | 52765 | 2171 |
| 766.537849329832 | 3.76 | PC 35:5 | C43H76NO8P |  | 31394 | 2272 |  | 32670 | 1566 |  | 27548 | 1617 |
| 786.596162070884 | 3.70 | PC 36:2 | C44H84NO8P |  | 332301 | 42679 |  | 307935 | 39582 |  | 333267 | 20787 |
| 782.567459651066 | 3.71 | PC 36:4 | C44H80NO8P |  | 2442170 | 230717 |  | 2443472 | 321903 |  | 2597066 | 264984 |
| 780.552364267460 | 3.73 | PC 36:5 | C44H78NO8P |  | 1801435 | 143097 |  | 1816263 | 210965 |  | 1840443 | 118984 |
| 778.536872623506 | 3.76 | PC 36:6 | C44H76NO8P |  | 615909 | 62108 |  | 570081 | 75612 |  | 544640 | 33033 |
| 816.617546364734 | 3.75 | PC 38:1 | C46H90NO8P |  | 370028 | 49389 |  | 378721 | 31241 |  | 402435 | 33614 |
| 814.609608776092 | 3.74 | PC 38:2 | C46H88NO8P |  | 170861 | 21879 |  | 168594 | 17393 |  | 177590 | 6470 |
| 812.607672459073 | 3.66 | PC 38:3 | C46H86NO8P |  | 33816 | 3434 |  | 33485 | 5520 |  | 33813 | 1988 |
| 808.577777539906 | 3.68 | PC 38:5 | C46H82NO8P |  | 93830 | 17590 |  | 86470 | 15658 |  | 96364 | 15413 |
| 804.553135052820 | 3.69 | PC 38:7 | C46H78NO8P |  | 105577 | 5994 |  | 116680 | 8859 |  | 124027 | 7277 |
| 840.616675153823 | 3.71 | PC 40:3 | C48H90NO8P |  | 191708 | 21357 |  | 193632 | 25555 |  | 206604 | 20337 |
| 828.554322797146 | 3.66 | PC 40:9 | C48H78NO8P |  | 16717 | 3602 |  | 15871 | 2887 |  | 17249 | 3531 |
| 868.668537596606 | 3.60 | PC 42:3 | C50H94NO8P |  | 9894 | 1698 |  | 10663 | 2419 |  | 10827 | 946 |
| 676.490970354433 | 2.68 | PE 31:1 | C36H70NO8P |  | 42668 | 3456 |  | 48001 | 4044 |  | 52411 | 6710 |
| 690.503581521432 | 2.69 | PE 32:1 | C37H72NO8P |  | 65849 | 7826 |  | 71288 | 5757 |  | 78890 | 10126 |
| 688.495405505980 | 2.69 | PE 32:2 | C37H70NO8P |  | 57528 | 6789 |  | 62587 | 4655 |  | 69485 | 7575 |
| 716.523574554207 | 2.63 | PE 34:2 | C39H74NO8P |  | 296988 | 38023 |  | 328520 | 33041 |  | 331272 | 22326 |
| 714.510715576371 | 2.64 | PE 34:3 | C39H72NO8P |  | 117803 | 15327 |  | 118849 | 11532 |  | 118259 | 3881 |
| 730.535156162020 | 2.62 | PE 35:2 | C40H76NO8P |  | 11546 | 972 |  | 11594 | 907 |  | 12597 | 1496 |
| 740.523546134838 | 2.60 | PE 36:4 | C41H74NO8P |  | 262333 | 30123 |  | 261533 | 24895 |  | 278279 | 28690 |
| 738.507977207778 | 2.62 | PE 36:5 | C41H72NO8P |  | 161674 | 18883 |  | 167705 | 16070 |  | 166551 | 9539 |

Data were acquired in positive mode. Adduct was [M+H+].
